# Computational assessment of feature combinations for pathogenic variant prediction

**DOI:** 10.1002/mgg3.214

**Published:** 2016-03-14

**Authors:** Eva König, Johannes Rainer, Francisco S. Domingues

**Affiliations:** ^1^ Center for BiomedicineEuropean Academy of Bozen/Bolzano (EURAC)Viale Druso 139100BolzanoItaly; ^2^ Affiliated Institute of the University of LübeckLübeckGermany

**Keywords:** Feature analysis, feature combination, gene features, GO annotation bias, pathogenic variant prediction, variant features

## Abstract

**Background:**

Although several methods have been proposed for predicting the effects of genetic variants and their role in disease, it is still a challenge to identify and prioritize pathogenic variants within sequencing studies.

**Methods:**

Here, we compare different variant and gene‐specific features as well as existing methods and investigate their best combination to explore potential performance gains.

**Results:**

We found that combining the number of “biological process” Gene Ontology annotations of a gene with the methods PON‐P2, and PROVEAN significantly improves prediction of pathogenic variants, outperforming all individual methods. A comprehensive analysis of the Gene Ontology feature suggests that it is not a variant‐dependent annotation bias but reflects the multifunctional nature of disease genes. Furthermore, we identified a set of difficult variants where different prediction methods fail.

**Conclusion:**

Existing pathogenicity prediction methods can be further improved.

## Introduction

High throughput sequencing technologies have evolved rapidly, providing new opportunities for investigating the genetic basis of disease in an affordable and efficient manner. Countless small‐ and large‐scale studies such as the 1000 Genomes Project (Abecasis et al. 2012), the HapMap Project (The International HapMap Consortium, 2003), or the Exome Sequencing Project have led to the discovery of millions of genetic variants. Consequently, there have been considerable efforts in characterizing human genetic variation and identifying the variants that have a functional impact (Ng and Henikoff 2006; Gnad et al. 2013). Of particular interest for investigating the genetic basis of heritable diseases are potentially pathogenic variants (Ferrer‐Costa et al. 2002; Ng and Henikoff 2006; Ye et al. 2007; Thusberg et al. 2011; de Beer et al. 2013). SIFT (Ng and Henikoff 2001) and PROVEAN (Choi et al. 2012), for example, compute scores based on multiple sequence alignments to assess whether substitutions are likely tolerated or deleterious. Several tools use statistical learning methods to estimate prediction rules from different types of features (Ferrer‐Costa et al. 2005; Adzhubei et al. 2010; Wang et al. 2012; Kircher et al. 2014; Niroula et al. 2015). Sequence conservation is a commonly used feature, since variants in highly conserved regions are usually not tolerated and can cause disease phenotypes (Wu and Jiang 2013). Other features, including different amino acid biochemical properties, sequence neighborhood, protein disorder, residue accessibility, and secondary structure information have been used by the methods PMUT (Ferrer‐Costa et al. 2005), SNAP (Bromberg and Rost 2007), FunSAV (Wang et al. 2012), and PolyPhen‐2 (Adzhubei et al. 2010). Some methods use a large number of features, for example, CADD (Kircher et al. 2014) combines 63 features to provide an estimate of deleteriousness. Recently, individual predictors have been combined into consensus predictors to further improve prediction performance. The most prominent examples are Condel (González‐Pérez and López‐Bigas 2011), CoVEC (Frousios et al. 2013), and PredictSNP (Bendl et al. 2014).

An important issue of these consensus predictors is the potential overlap of training and validation data. Performance evaluation on variants that have been used to train the input predictors may lead to an overoptimistic assessment of the consensus predictor, a problem described as circularity (Grimm et al. 2015). In particular, Grimm et al. investigated the effects of two types of circularity: type 1 circularity arising from the overlap of variants in the training and validation data, type 2 circularity resulting from distinct variants in these sets that are located in the same genes.

Most features used in prediction methods are variant specific, as they capture a property of the mutated amino acid or its position in the protein. Few methods have attempted to incorporate gene‐specific features, which may only aid classification in the combination with variant features, since gene features necessarily have the same values for pathogenic and benign variants in the same gene. SNPs&GO (Calabrese et al. 2009) and PON‐P2 (Niroula et al. 2015) incorporate a feature based on Gene Ontology (GO) annotations in their prediction scores, while SySAP (Huang et al. 2012) and SuSPect (Yates et al. 2014) include gene features derived from protein interaction network measures. Yet, to the best of our knowledge, the potential performance gain for variant prediction by integrating different gene features has not been assessed systematically.

Even though a great number of prediction methods have been developed in recent years, two main challenges remain. First, investigate if existing methods can be further improved, in particular by integrating gene features, while avoiding circularity. Second, identify and characterize difficult cases where individual methods tend to mispredict and assess the extent of common weaknesses.

In this study, we perform an analysis of 16 variant‐ and 12 gene‐based features, including current methods for predicting variation effect, and assess their individual and combined contribution to pathogenicity prediction. We derive sets of features that archive the highest prediction accuracy and analyze the constituting features in more detail. Finally, we derive a set of difficult variants where different methods fail, which might be useful for future method development.

## Materials and Methods

### Ethical compliance

Given that only data from public databases and HGMD^®^ Professional was analyzed in this study, an ethics committee approval was not required.

### Polymorphism datasets

Sets of pathogenic and benign single amino acid polymorphisms (SAPs) were created based on the corresponding missense single‐nucleotide polymorphisms (SNPs). The analysis was restricted to missense SNPs, since many properties are not defined for other types of small variations. Pathogenic missense SNPs were taken from entries with the “mutation class” “disease mutation (DM)” in the Human gene mutation database (HGMD^®^ Professional) from BIOBASE Corporation (Stenson et al. 2014) version 2014.03 or with the “clinical significance” “pathogenic” in the ClinVar database (Landrum et al. 2014) version 20140929. Pathogenic SNPs were only included, if they were either not observed in the phase 3 calls of the 1000 Genomes Project (Abecasis et al. 2012) or were observed with an allele frequency smaller than 0.01. This restriction ensured a reduction of false SNPs in the pathogenic set. Benign missense SNPs were selected from the phase 3 1000 Genomes calls under the constraint that they have an allele frequency greater than 0.01 and that they are not listed as “DM” in HGMD^®^ version 2014.03 or as “pathogenic” in ClinVar version 20140929. To avoid gender‐specific effects on the X and Y chromosomes, only SNPs on the autosomes were selected. A total of 90% of the pathogenic and benign SAPs were randomly selected to constitute a *training* set used for feature selection and model training, while the remaining 10% constitute the *validation* set used for the final model evaluation. To avoid type 2 circularity (Grimm et al. 2015), all SAPs from one gene were assigned either to the *training* or to the *validation* set.

Preliminary analysis on the *training* set showed that PON‐P2 (Niroula et al. 2015) and Condel (González‐Pérez and López‐Bigas 2011) are excellent predictors for pathogenicity. To avoid type 1 and 2 circularity, we removed any SAP from the *training* and *validation* sets for which either the SAP itself or its gene overlapped with train or test data used in the development of PON‐P2. Unfortunately, the training sets used in the weight tuning of Condel and in its two constituting predictors FatHMM (Shihab et al. 2013) and MutationAssessor (Reva et al. 2011) almost completely contain all currently available variants and their removal would have precluded any meaningful analysis. To avoid spurious results due to circularity, we excluded Condel, PolyPhen‐2 (Adzhubei et al. 2010), and CADD (Kircher et al. 2014) from the main analysis. An analysis including these methods is available in Data S1. The final number of SAPs in the *training* set are pathogenic = 14,033 (in 1,753 genes), benign = 15,574 (in 6,120 genes); and in the *validation* set pathogenic = 2,085 (in 241 genes), benign = 2,351 (in 856 genes). The overlap of genes including both pathogenic and benign variants for both sets is shown in Fig. S1. The overlap of the *training* and *validation* datasets to the existing datasets ExoVar (Li et al. 2013), HumVar (Adzhubei et al. 2010), SwissVar (Mottaz et al. 2010), and VariBench (protein tolerance dataset 1) (Nair and Vihinen 2013) is shown in Fig. S2. The handling of variants with missing data is described in Data S1. The complete datasets are available upon request to the authors, if a valid HGMD Professional license is provided. Without a license, the datasets can be reduced to include only SAPs from 1000 Genomes and ClinVar.

### Features

Twenty‐eight features were analyzed for their discriminative power in SAP classification. Of these, 16 are variant features, whose values depend on properties of the nucleotide or amino acid change, or on its position in the protein. The remaining 12 are gene features, whose values exclusively depend on characteristics of the genes in which the SAPs are located (see Table 1). In the description below, feature names are highlighted in italics when they appear for the first time.

**Table 1:** The twenty‐eight features used in this study

Feature	Class	Type 1	Type 2	Description	Reference
PON‐P2	Numeric	Variant	TPS	PON‐P2 score	Niroula et al. (2015)
SIFT	Numeric	Variant	RPS	SIFT score	Ng and Henikoff (2001)
PROVEAN	Numeric	Variant	RPS	PROVEAN score	Choi et al. (2012)
Grantham	Numeric	Variant	RPS	Grantham score	Grantham (1974)
GERP	Numeric	Variant	RPS	GERP conservation score, computed on 37 eutherian mammals	Cooper et al. (2005)
evolutionary.rate	Numeric	Variant	Raw feature	Residue evolutionary rate computed with rate4site on PhylomeDB alignments	Pupko et al. (2002), Huerta‐Cepas et al. (2014)
disordered.region	Numeric	Variant	Raw feature	Disordered region value between 0 and 1, computed with SPINE‐D. Ordered: val < 0.5, disordered: val ≥ 0.5	Zhang et al. (2012)
accessibility	Numeric	Variant	Raw feature	Residue accessibility value between ‐5 and 95, computed with SCRATCH‐1D. Low values correspond to buried residues, high values to exposed residues	Cheng et al. (2005), Magnan and Baldi (2014)
secondary.structure.3	Categorical	Variant	Raw feature	Secondary structure prediction, 3 class, computed with SCRATCH‐1D	Cheng et al. (2005), Magnan and Baldi (2014)
secondary.structure.8	Categorical	Variant	Raw feature	Secondary structure prediction, 8 class, computed with SCRATCH‐1D	Cheng et al. (2005), Magnan and Baldi (2014)
PfamA	Categorical	Variant	Raw feature	1 if the variant is in a PfamA domain, 0 else	Finn et al. (2004)
AAindex.polarity	Numeric	Variant	Raw feature	AAindex GRAR740102 (Polarity) from section AAindex1	Kawashima et al. (2008)
AAindex.hydropathy	Numeric	Variant	Raw feature	AAindex KYTJ820101 (Hydropathy) from section AAindex1	Kawashima et al. (2008)
AAindex.volume	Numeric	Variant	Raw feature	AAindex GRAR740103 (Volume) from section AAindex1	Kawashima et al. (2008)
AAindex.composition	Numeric	Variant	Raw feature	AAindex KH900101 (AA composition of total proteins) from section AAindex1	Kawashima et al. (2008)
AAindex.net.charge	Numeric	Variant	Raw feature	AAindex KLEP840101 (Net charge) from section AAindex1	Kawashima et al. (2008)
protein.age	Numeric	Gene	Raw feature	Protein age computed with ProteinHistorian	Capra et al. (2012)
paralog.id	Numeric	Gene	Raw feature	Maximum paralog identity value of gene, 0 if the gene has no paralog	Cunningham et al. (2015)
paralog.nr	Numeric	Gene	Raw feature	Number of human paralog genes	Cunningham et al. (2015)
mouse.orth.id	Numeric	Gene	Raw feature	Maximum mouse ortholog identity value of gene, 0 if the gene has no mouse ortholog	Cunningham et al. (2015)
mouse.orth.nr	Numeric	Gene	Raw feature	Number of mouse ortholog genes	Cunningham et al. (2015)
GO.BP	Numeric	Gene	Raw feature	Number of GO BP annotations of a gene with information content greater 2 considering all evidence codes and disregarding children nodes	The Gene Ontology Consortium (2015)
expression	Numeric	Gene	Raw feature	Fraction of tissues in which this gene is expressed at a threshold	Kolesnikov et al. (2015)
degree	Numeric	Gene	Raw feature	Degree of gene on the mentha network	Calderone et al. (2013)
centrality	Numeric	Gene	Raw feature	Alpha centrality of gene on the mentha network	Calderone et al. (2013)
betweenness	Numeric	Gene	Raw feature	Betweenness of gene on the mentha network	Calderone et al. (2013)
gene.length	Numeric	Gene	Raw feature	Gene length in base pairs	Cunningham et al. (2015)
protein.length	Numeric	Gene	Raw feature	Protein length in base pairs	Cunningham et al. (2015)
PolyPhen‐2^a^	Numeric	Variant	TPS	PolyPhen‐2 score	Adzhubei et al. (2010)
Condel^a^	Numeric	Variant	TPS	Condel 2.0 score (weighted average of FatHMM and MutationAssessor)	González‐Pérez and López‐Bigas (2011)
CADD^a^	Numeric	Variant	TPS	CADD phred score	Kircher et al. (2014)

TPS, trained prediction score; RPS, rule prediction score.

a Not used as features in the main analysis due to circularity.

#### Variant features

*SIFT* (Ng and Henikoff 2001), PolyPhen‐2 (Adzhubei et al. 2010), *GERP* (Cooper et al. 2005), and corresponding gene ids were queried from the Ensembl database version 75 (Cunningham et al. 2015) using the Dintor software suit (Weichenberger et al. 2015). Condel 2.0 scores db‐version 05 (González‐Pérez and López‐Bigas 2011) (weighted average of FatHMM (Shihab et al. 2013) and MutationAssessor (Reva et al. 2011)), *PON‐P2* scores (Niroula et al. 2015), *PROVEAN* scores v1.1.3 (Choi et al. 2012), and CADD phred scores v1.2 (Kircher et al. 2014) were queried via the respective web servers. *Grantham* scores (Grantham 1974) were extracted from the CADD output. To obtain a conservation measure on the protein residue level, an evolutionary rate feature (*evolutionary.rate*) was computed with the rate4site program version 3.0.0 (Pupko et al. 2002) on multiple sequence alignments obtained for each protein from the PhylomeDB database v4 (Huerta‐Cepas et al. 2014). Rate4site computes an evolutionary rate for every residue position in the alignment. The Needleman–Wunsch algorithm from the EMBOSS‐6.6.0 program (Rice et al. 2000) was used to map the sequences in PhylomeDB to the reference amino acid sequence obtained from Ensembl version 75. Disordered region (*disordered.region*) values for the reference amino acid at all positions in all proteins were computed with the Spine‐D program version 2.0 (Zhang et al. 2012) and the values at the corresponding positions were mapped to the SAPs. Values smaller than 0.5 indicate ordered residues, while values greater than or equal to 0.5 indicate disordered residues. In the same manner, residue accessibility (*accessibility*) and secondary structure according to the three and eight class definition (*secondary.structure.3* and *secondary.structure.8*, respectively) were computed with the SCRATCH‐1D program version 1.0 (Cheng et al. 2005; Magnan and Baldi 2014) on the reference amino acids. Regions with PfamA domains were obtained from Pfam 27.0 (Finn et al. 2004). The feature *PfamA* is a binary variable indicating whether the variant is located within a PfamA domain or not. Five amino acid indices were selected from the AAindex database (section AAindex1) (Kawashima et al. 2008) to represent distinct biochemical properties of amino acids. For each SAP, the absolute difference in the respective AAindex value for the original and mutated amino acid was calculated, creating the five features *AAindex.polarity* (GRAR740102), *AAindex.hydropathy* (KYTJ820101), *AAindex.volume* (GRAR740103), *AAindex.composition* (KH900101), and *AAindex.net.charge* (KLEP840101). We name PolyPhen‐2, Condel, PON‐P2, and CADD “trained prediction scores” (TPS), as their values are determined by trained statistical models. Since the training data of PolyPhen‐2, Condel, and CADD overlap with our *training* set, we do not use these scores as possible input features for our models in the main analysis (see Data S1 for an analysis with all TPS). We name SIFT, PROVEAN, GERP, and Grantham “rule prediction scores” (RPS), where the first three are computed from multiple sequence alignments and the latter reflects biochemical properties of the amino acid change. These features are not affected by circularity, since no machine learning was performed. We call the remaining variant features and the gene features described below as “raw features”. The selection of features aims to cover different strategies that have been proposed for predicting variant effect, including basic biochemical properties and established prediction methods. The selected features rely on the properties of the substituted nucleotide or residue or their position, however, PON‐P2 also takes gene annotations into account.

#### Gene features

A conservation measure on the gene level (*protein.age*) was obtained with ProteinHistorian version 1.0 using default parameters (Capra et al. 2012). Human gene paralogs and mouse orthologs were downloaded from Ensembl 77. A human gene has none, one, or multiple paralogous and orthologous genes, each annotated with a confidence of zero or one and an identity value between 0 and 100, which reflects the similarity of the paralogous or orthologous gene with the target gene. Two paralog and ortholog features were defined, considering only paralogs and orthologs with a confidence of one: the number of paralogous genes (*paralog.nr*), and their maximum identity value (*paralog.id*), which was assigned zero for genes that did not have any paralogous genes. Mouse ortholog features were computed accordingly (*mouse.orth.nr* and *mouse.orth.id*). “Biological process” (BP) annotations in the Gene Ontology (GO) database version 2014.11 (The Gene Ontology Consortium, 2015) were used to obtain a measure for gene multifunctionality corresponding to the gene's number of BP annotations and thus the number of processes it is involved in. Specifically, the feature *GO.BP* was computed with the Dintor software suit (Weichenberger et al. 2015) as the number of GO BP annotations of a gene, considering all evidence codes (see Data S1 for command line parameters). To obtain a nonredundant set of GO annotations, two filtering steps were performed prior to the counting. First, terms with an information content (IC) smaller 2.0 were excluded, since these are nonspecific terms that add little information. The threshold of 2.0 was determined heuristically. Examples for excluded terms are “cellular metabolic process” (IC = 0.48) and “oxidation–reduction process (IC = 1.52)”. More specific terms like “leukocyte activation” (IC = 2.03) or “complement‐dependent cytotoxicity” (IC = 5.87) were included. Second, if a gene was annotated with a term and its child terms, only the parent term was counted, since its child term only represents a specification of the GO term and not a distinct BP. For example, the GO term “mitochondrial respiratory chain complex assembly” has the child “mitochondrial respiratory chain complex I assembly”. The IC filtering was performed prior to the removal of the children. The gene expression dataset E‐MATB‐1733 containing expression values for 19,021 human genes in 27 tissues was downloaded from the ArrayExpress database (Kolesnikov et al. 2015). The feature *expression* is the fraction of tissues that were expressed at a 3.5 threshold, which is the mean first quartile value of the distribution of tissue expression at the gene level. A network with 157,962 human protein–protein interactions was downloaded from mentha on 2014‐11‐24 (Calderone et al. 2013). To restrict the network to interactions with high confidence, interactions with a confidence score below 0.126 (the first quartile value of the distribution of the confidence scores of the whole network) were discarded. The filtered network consisted of 140,289 interactions. On this network, three features (*degree*,* centrality*, and *betweenness*) were computed for each gene with the igraph package version 0.6.5‐2 (Csardi and Nepusz 2006) in the statistical programming language R version 3.1.0. Finally, the features *gene.length* (the gene length in base pairs including introns) and *protein.length* (the number of amino acids of the corresponding protein) were retrieved from Ensembl.

### Clustering of features

Hierarchical clustering on all numeric features was performed on the absolute values of the features’ Pearson correlation, thus positively and negatively correlated features cluster together. The categorical features PfamA, secondary.structure.3, and secondary.structure.8 were excluded from the analysis. The optimal number of clusters and their quality was determined as the cluster composition that maximizes the clusters’ mean silhouette values (Rousseeuw 1987) (R package hopach_2.28.0). A feature's silhouette value lies between −1.0 and 1.0: a value of 1.0 indicates that the feature is optimally clustered, while a value of −1.0 indicates that the assignment to a different cluster would be more appropriate. Clusters with high silhouette values thus correspond to high cluster quality and include correlated features distinct from features in other clusters.

### Decision trees, logistic regression, and random forest models

To determine which of the 28 features characterize pathogenic and benign SAPs, a classification problem was defined. The binary response variable “class” encodes the true variant status of pathogenic (1) or benign (0), and the 28 features are possible predictors or input variables. To obtain an interpretable set of rules, decision trees were computed with the R package rpart 4.1‐9 using default parameters. To assess feature importance and to select sets of features for prediction, stepwise forward selection was performed with both logistic regression (R package stats) and random forests (R package party 1.0‐20 (Hothorn et al. 2006; Strobl et al. 2007, 2008). The model was trained once on all features on the full *training* set. For logistic regression, the features were then ordered based on the *P*‐value of the Wald *z*‐statistic, which tests whether the effect of a feature is statistically significant on the outcome *class*. For random forests, the features were ordered based on their importance, as computed by the *varimp* function of the *party* package. This implementation reflects feature importance accurately even in the presence of feature correlation (Strobl et al. 2008). Starting with the first feature in the ordered list, the next feature was added sequentially, resulting in one additional feature per step. To evaluate the model with the current feature set, 300 and 100 iterations of 5‐fold cross validation (CV) were computed for logistic regression and random forests, respectively. For each iteration in the CV, SAPs were randomly divided into subsets for training and testing, such that all SAPs from one gene were either in the training or in the testing subset. The train and test errors were computed as the residual sum of squares and the misclassification rate for logistic regression and random forests, respectively. For the predictive feature sets derived on the *training* set, logistic regression models were computed on the *validation* set. The area under the curve (AUC) was computed for each logistic regression model and SIFT, PON‐P2, and PROVEAN for comparison. The AUCs were ordered based on size and a Bootstrap test (pROC package (Robin et al. 2011), 5000 permutations) was performed to test for significant differences in AUC size of each method with the next best method. All analyses were performed with the R statistical software version 3.2.1.

## Results

### Feature comparison

**Figure 1:**
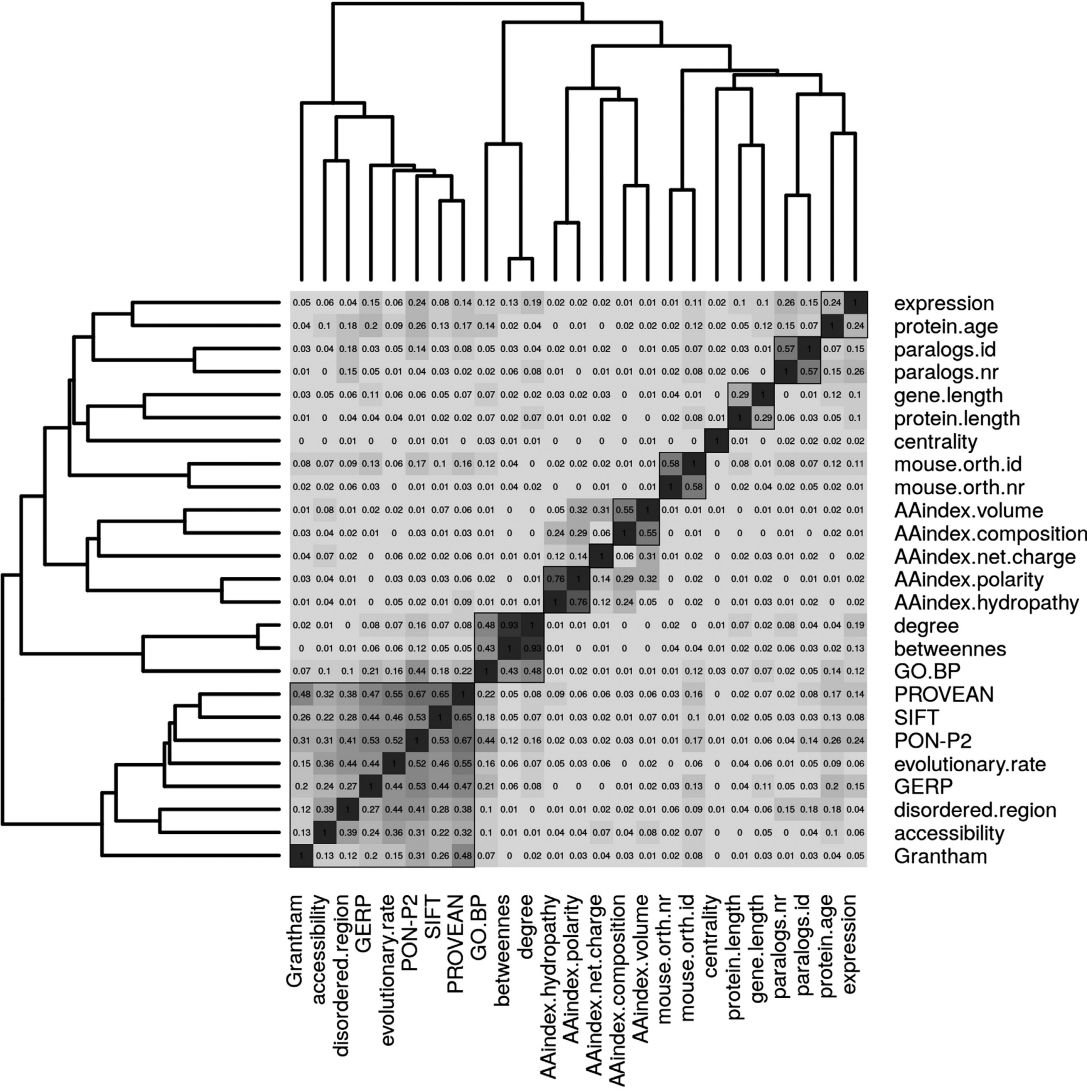
Hierarchical clustering of all features (except PfamA, secondary.structure.3, and secondary.structure.8) on their absolute Pearson correlation values. In each cell, the rounded absolute correlation value is given, the higher the value, the darker the corresponding cell. Black squares correspond to the 10 clusters that were found to maximize the mean silhouette values.

Prior to the selection of the best predictive features, we performed an unsupervised hierarchical cluster analysis to evaluate between‐feature correlations (see Fig. 1 and Table S2). Silhouette width analysis showed that the optimal number of clusters was 10. The cluster with best quality (highest silhouette width) corresponds to the amino acid index features, polarity and hydropathy with a silhouette width value of 0.69. The largest, but only sixth best cluster with a silhouette width of 0.28 includes all RPSs, PON‐P2, and the raw scores disordered.region, evolutionary.rate, and accessibility. The correlation of these features is expected, as these methods include evolutionary models for estimating residue conservation and for assessing deleterious substitutions. In general, there is low correlation between features in different clusters with few exceptions. In this respect, we observe a correlation of 0.44 between GO.BP and the PON‐P2 method, since the latter includes a GO‐derived feature as one of its inputs. Further, the amino acid index features that are not clustered together have correlation values between 0.05 and 0.32.

### Feature selection

**Figure 2:**
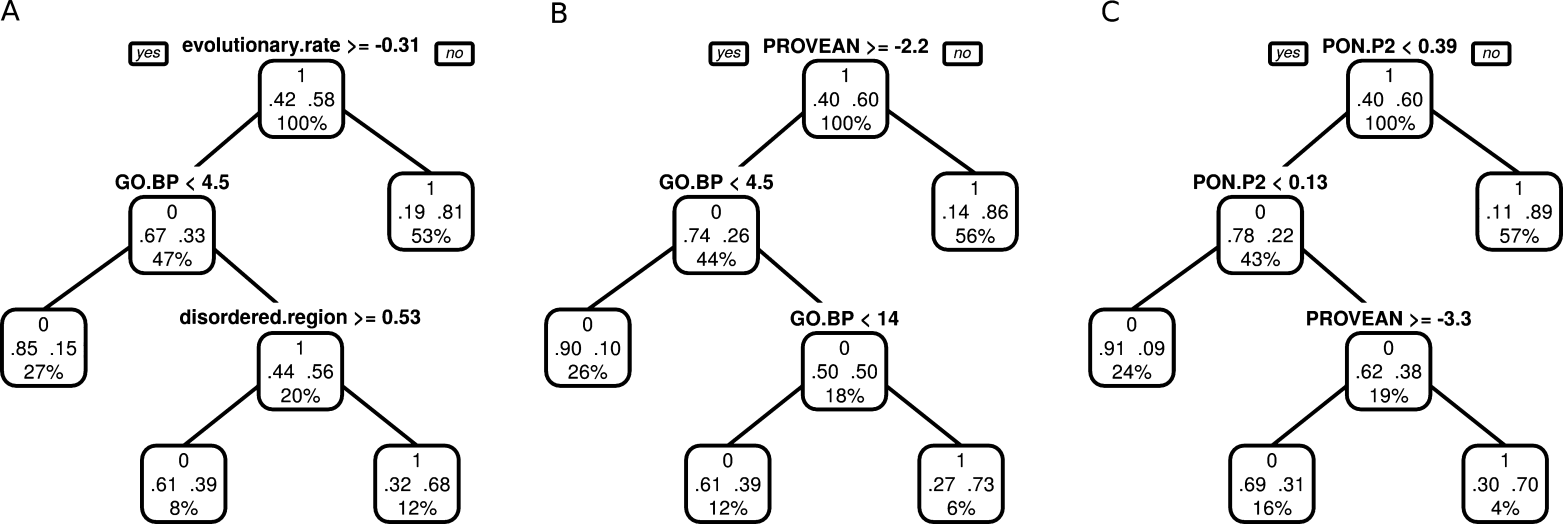
Decision trees computed on the *training* set using different input features. Each tree node has three rows: the upper row contains the decision made in this node with 0 = benign and 1 = pathogenic; the second row shows the fraction of single amino acid polymorphisms (SAPs) classified at this node as benign (left) and pathogenic (right); the third row shows the percentage of all input SAPs that are classified at this node. Starting from the root node, at each node, the left child is traversed if the condition evaluates to true and the right child is traversed if the condition evaluates to false. (A) Tree computed on raw features, excluding rule prediction scores (RPSs) and PON‐P2. (B) Tree computed on raw features and RPSs, excluding PON‐P2. (C) Tree computed on all features.

Next, we computed decision trees on the *training* set to get an initial overview of the most important features discriminating pathogenic from benign variants. To obtain a biologically interpretable result, we first excluded RPSs and the TPS PON‐P2 from the analysis and sequentially added RPS and PON‐P2 back to evaluate whether predictions improve. Using only raw scores as input resulted in a classification tree with the variant features evolutionary.rate, and disordered.region, and the gene feature GO.BP (see Fig. 2A). Inclusion of RPS resulted in a tree with PROVEAN and GO.BP (see Fig. 2B) and additionally including PON‐P2 resulted in a tree with the features PON‐P2 and PROVEAN (see Fig. 2C). Inclusion of RPS features and PON‐P2 improved the predictive power of the respective classification tree. The error rate for the tree based only on raw features was 0.21, it decreased to 0.17 after inclusion of RPS and to 0.14 after inclusion of all features.

**Figure 3:**
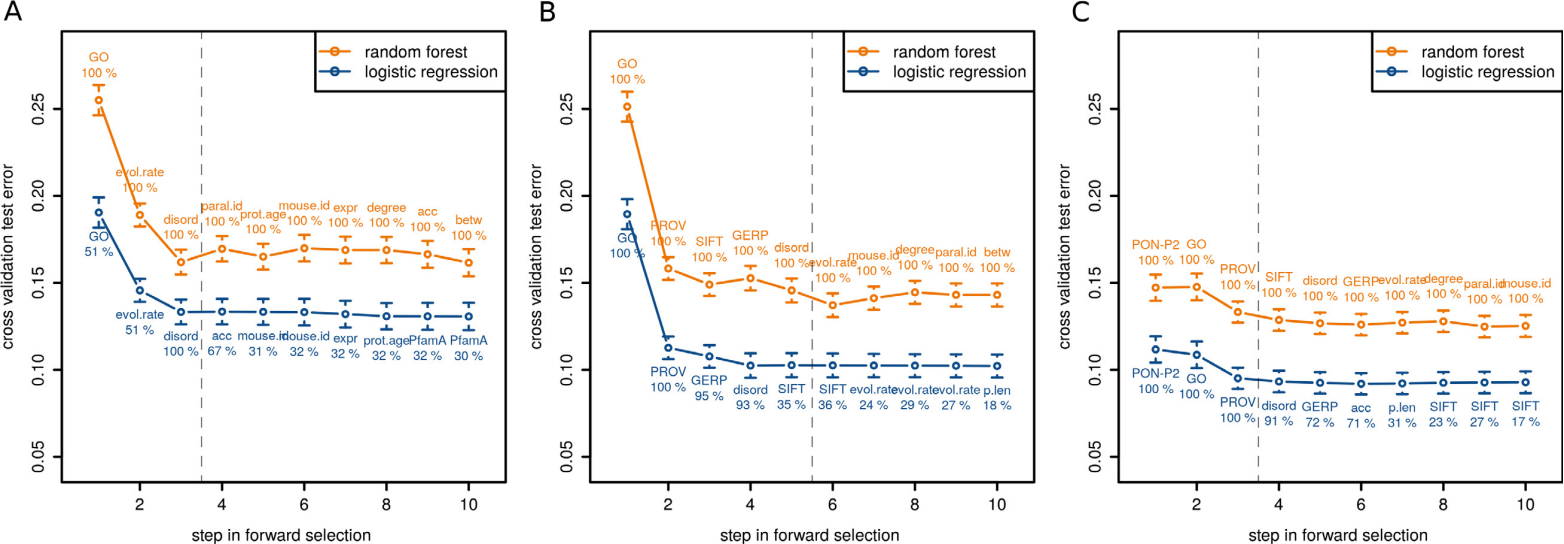
Cross‐validation (CV) test error of stepwise forward selection with random forest and linear regression. Points correspond to the mean test error from all CV iterations with error bars. The label corresponds to the feature that was added in this step and the number below indicates the percentage of CV iterations in which this feature was selected. Labels are printed above and below the points for random forest and linear regression, respectively. The vertical dashed line shows the cutoff for feature selection. Only the first 10 steps of the forward selection are shown. GO = GO.BP, evol.rate = evolutionary.rate, disord = disordered.region, paral.id = paralog.id, acc = accessibility, prot.age = protein.age, mouse.id = mouse.orth.id; expr = expression, betw = betweeness, PROV = PROVEAN, p.len = protein.length, Grant = Grantham. (A) Stepwise forward selection on raw scores, excluding rule prediction scores (RPS) and PON‐P2. (B) Stepwise forward selection on raw scores and RPSs, excluding PON‐P2. (C) Stepwise forward selection on all features.

As an alternative approach to determine the best combination of discriminative features, we employed a forward feature selection strategy based on logistic regression and random forests. Again, we used a multistep approach, that is, first excluding RPS and PON‐P2 from the analysis and sequentially including them to evaluate differences in classification performance. As in the classification tree approach, both logistic regression and random forest identified the features GO.BP, evolutionary.rate, and disordered.region as the most predictive among all raw features (see Fig. 3A). After the inclusion of these features, the random forest error rate increased, therefore only the first three features were selected to form the predictive feature set 1 (PFS1). In 49% of the logistic regression CV iterations, evolutionary.rate was selected as first and GO.BP as second feature. Next, we included the RPS to the input features and repeated the analysis (see Fig. 3B). Both random forest and logistic regression selected GO.BP, PROVEAN, SIFT, GERP, and disordered.region as the five most important features. Determining a good cutoff for feature selection was less straightforward in this case, but additional analysis (see Fig. S3) showed that the first five features constitute the best performing subset and were thus selected to form predictive feature set 2 (FPS2). Then, we performed the feature selection analysis on the full feature set including PON‐P2. The most predictive features for both logistic regression and random forests were PON‐P2, GO.BP, and PROVEAN (see Fig. 3C), which were combined into the predictive feature set 3 (PFS3).

To summarize, we defined three sets of features capable to discriminate pathogenic from benign variants. We found a good agreement for best discriminative features among the different feature selection methods. PFSs that include scores from pathogenic variant prediction methods in combination with raw features dramatically decreased the CV error rate. PON‐P2 was chosen for PFS3, even though there is no overlap of our *training* set with the PON‐P2 train and test data. Importantly, the gene feature GO.BP was found to be among the most discriminative features in all analyses and was included in all PFSs.

### Analysis of selected features and GO annotation bias

**Figure 4:**
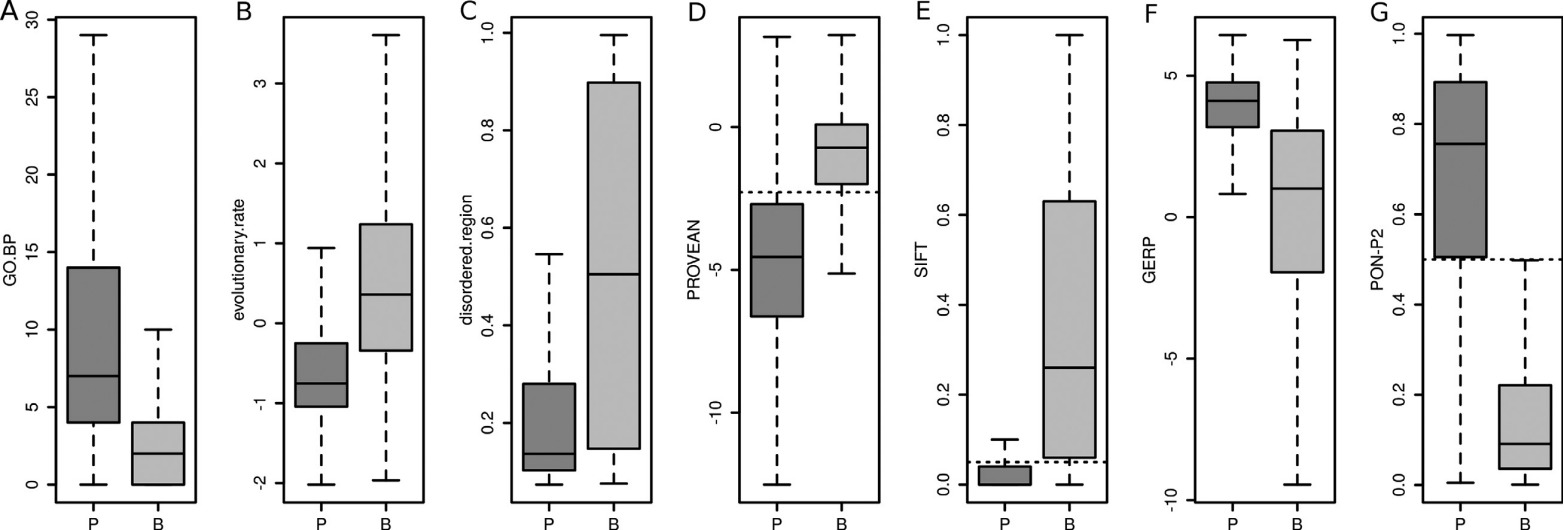
Box‐and‐whisker plots of the seven features constituting the predictive feature sets 1, 2, and 3 on the *training* set in pathogenic (P) and benign (B) single amino acid polymorphisms. The boxes show the first and third quartile of the distributions with their median; the whiskers extend to 1.5 times the interquartile range. For PROVEAN, SIFT, and PON‐P2, the classification thresholds estimated by the developers are shown as dashed horizontal lines. (A) GO.BP. (B) evolutionary.rate, (C) disordered.region. (D) PROVEAN. (E) SIFT. (F) GERP. (G) PON‐P2.

**Figure 5:**
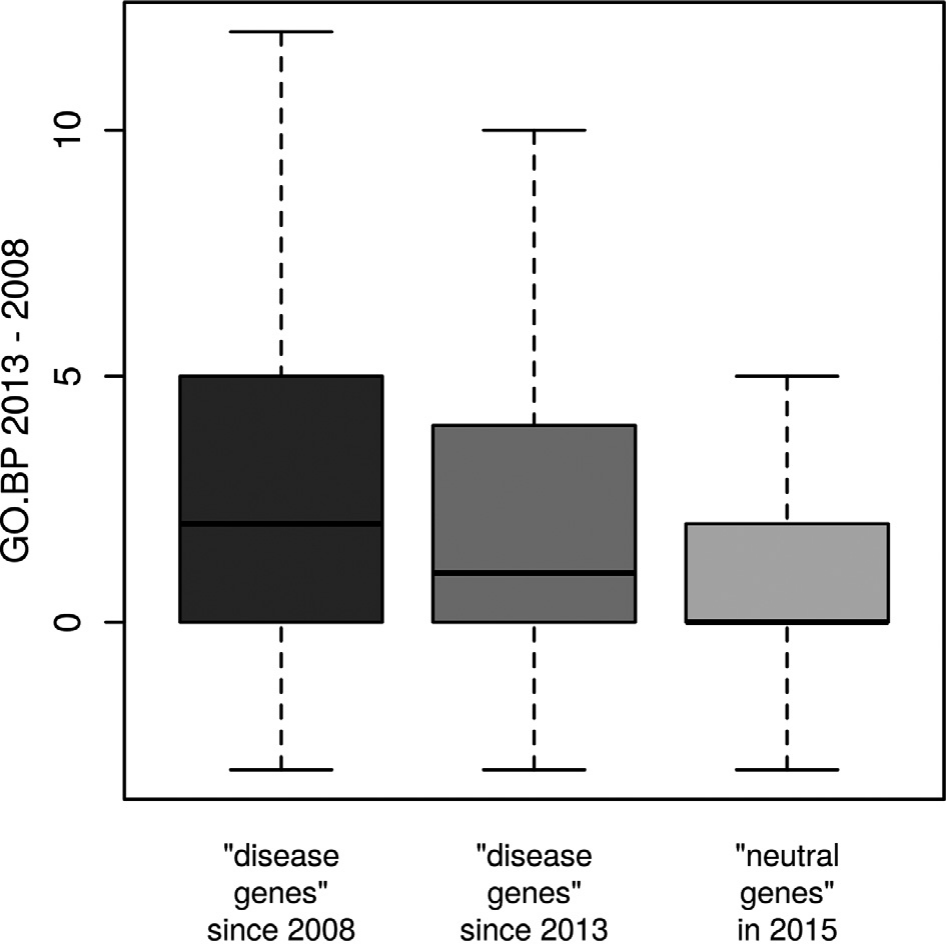
Increase in Gene Ontology (GO) biological process (BP) annotations from 2008 to 2013 for 957 genes for which the first disease variant has been reported in HGMD in 2008 (left, first group), for 349 genes for which the first disease variant has been reported in HGMD in 2013 (middle, second group), and for 13,001 genes that have no pathogenic variant reported in HGMD (right, third group).

To better understand the impact of the seven features selected in PFS1, PFS2, and PFS3, we analyzed them in more detail (see Fig. 4). Genes of pathogenic variants had more than three times as many GO BP annotations than genes of benign variants (see Fig. 4A). This might indicate that genes related to disease tend to be involved in multiple processes. However, an important question is to what extent the GO.BP feature truly measures the level of gene multifunctionality and to what extend it represents an annotation bias. Certain genes and BPs have been studied more intensively than others, for example, due to their involvement in disease, and thus have more annotations associated. For our analysis, it is important to determine if reports of disease‐associated variants in genes lead to follow‐up studies on these genes, which then result in an increase in GO BP annotations. To address this question, we compared GO releases 2013.03 and 2008.01, which was the oldest easily assessable GO version. We determined all genes for which the first disease variant had been added to HGMD version 2014.03 in 2008 (*n* = 957, first group), all genes for which the first disease variant had been added to HGMD version 2015.01 in 2013 (*n* = 349, second group), and all protein‐coding genes which did not have a pathogenic variant reported in HGMD version 2015.01 (*n* = 13,001, third group), and calculated the difference of GO.BP between the 2013 and 2008 data for these three groups of genes. The first group of genes was known to harbor disease variants in this time period, while for the second group, this was only discovered afterward. If a variant‐dependent GO bias existed, we would expect the increase of annotations in the first group to be larger than in the second group. However, even though the mean is higher in the first group than in the second group (see Fig. 5), a one‐sided student's *t*‐test showed that the difference in mean between the two distributions was not significant at a 5% threshold (*P* = 0.1). Therefore, in the time period from 2008 to 2013, knowledge of disease variants in genes did not lead to a significant increase in their number of GO BP annotations compared with genes for which no disease variants were known in this period. For the second and third group, no pathogenic variants were known in these genes until 2013. That is, neither group of genes was more intensively studied due to knowledge of pathogenic variants between 2008 and 2013. Yet, the increase in GO BP annotations in the second group is significantly larger than the increase in the third group (one‐sided student's *t*‐test, *P* = 1.5 × 10^−5^) (see Fig. 5). Since the second group comprises genes that actually harbor pathogenic variants, while the third group does not (given the state of knowledge in 2015), these results indicate that multifunctionality as measured by the GO.BP feature is a true characteristic of disease genes.

For another feature of PFS1, evolutionary.rate, pathogenic SAPs had lower values than benign SAPs, which agrees well with previous findings that changes at evolutionary conserved sites tend to be deleterious (Ng and Henikoff 2003; Cooper et al. 2005) (see Fig. 4B). Pathogenic SAPs further had lower disordered.region values, that is, they tend to lie in regions with more stable conformations, while benign SAPs tend to lie in more disordered regions (see Fig. 4C). Using the threshold of 0.5 to classify the residues as ordered or disordered, these results agree very well with a previous analysis (Vacic et al. 2012) and indicate that SAPs in ordered regions interrupt protein structure, activity, and stability. In contrast, by using disordered region definitions from the DisProt database, Ye et al. (2007) found that 112 of 114 disease‐associated SAPs lie in disordered regions. Similarly, Huang et al. (2010) found that the disorder of two amino acids ahead of the SAP was the second most important predictor for deleteriousness. Pathogenic and benign SAPs were well separated by PROVEAN and SIFT scores according to the empirical thresholds proposed by the respective developers (see Figs. 4D and E). Pathogenic variants had GERP values that were about twice as high as those of benign variants (see Fig. 4F), which, like evolutionary.rate, supports previous findings that variants in conserved regions tend to be deleterious. The PON‐P2 values in pathogenic and benign SAPs separated even more clearly than the PROVEAN values (see Fig. 4G). The distribution of values in pathogenic and benign SAPs for all remaining features is shown in Fig. S4.

### Combination of variant and gene‐based features improves variant classification

**Figure 6:**
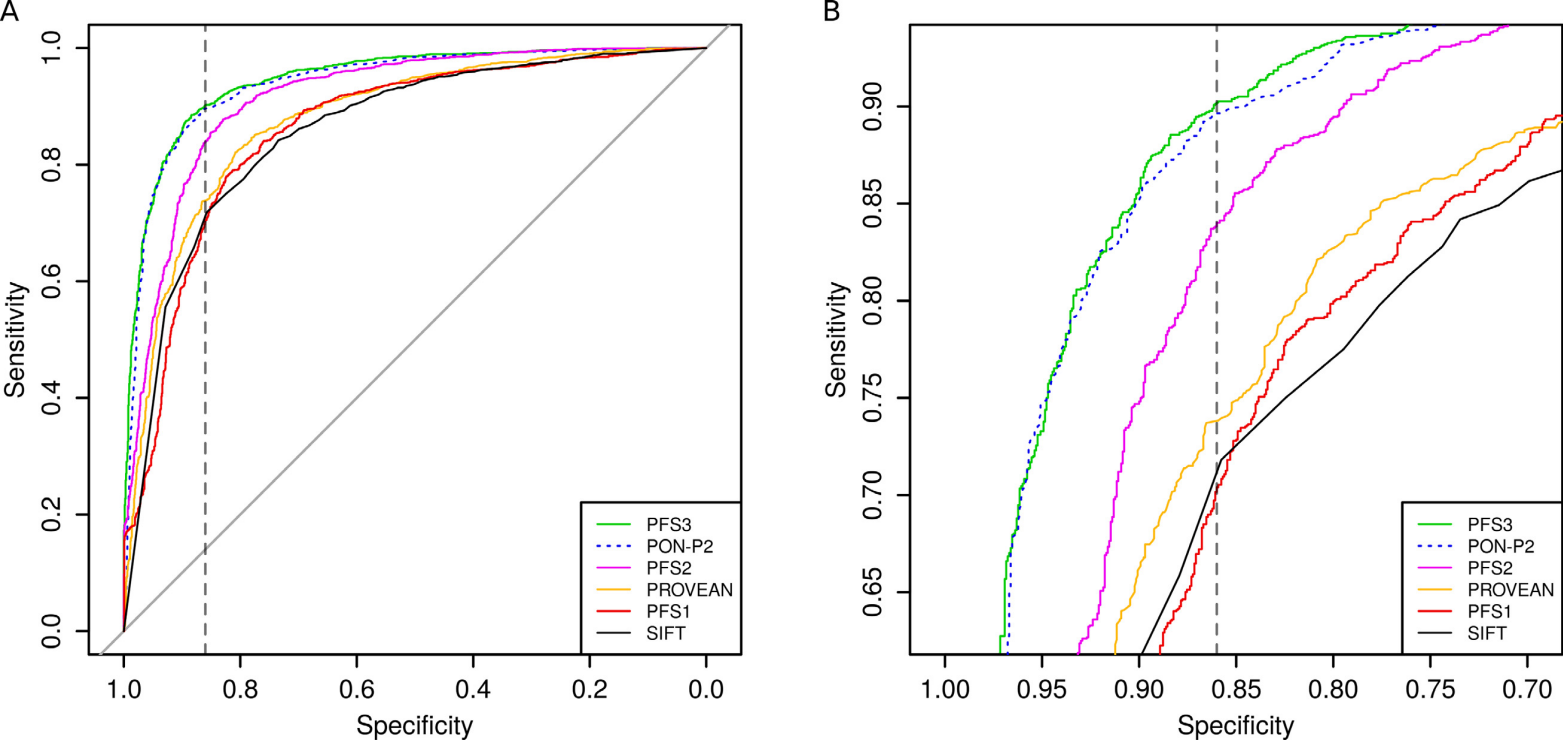
Receiver operating characteristic (ROC) curves showing specificity versus sensitivity of the logistic regression models and prediction scores at different thresholds on the *validation* set. In the legend, models are ordered according to their AUC values. One line is dotted to improve visibility. (A) Full ROC curve. The vertical dashed line at 0.86 corresponds to the specificity of PON‐P2 as estimated by the developers. (B) Same data as in (A), zoomed into the region where the lines of the ROC curve intersect the specificity threshold of 0.86.

Next, we evaluated the performance of logistic regression classifiers built on the three PFS on the independent *validation* data set. For each PFS, we first trained a logistic regression classifier using the *training* data set, obtained predictions for the *validation* set, and calculated receiver operating characteristic (ROC) curves. To enable comparisons with existing pathogenic variant prediction algorithms, ROC curves were also computed for SIFT, PON‐P2, and PROVEAN on the *validation* set (see Fig. 6A). For each method, we calculated the AUC, ordered the AUCs by size, and tested for significance at a 5% threshold between the AUC values of a method and the next best (see Table 2). For the logistic regression models of PFS1, PFS2, and PFS3, we determined the cutoff that maximized the Matthew's correlation coefficient (MCC). For PON‐P2, PROVEAN, and SIFT cutoffs were used as proposed by the developers of the respective method. Using these cutoffs, we computed sensitivity, specificity, accuracy, positive predictive value, negative predictive value, and MCC for all methods (see Table 2). Logistic regression based on PFS3 had the best performance with an AUC of 0.95, which was significantly larger than the AUC of the next best performing method PON‐P2. PFS3 further had the highest accuracy and MCC of all predictors. PON‐P2 performed significantly better than the logistic regression based on PFS2 and had the second highest AUC and MCC. After PFS2, the next best method was PROVEAN, followed by the logistic regression based on PFS1, and SIFT (see Table 2). The performance of the established prediction methods Condel, PolyPhen‐2, and CADD has also been investigated, but the results for these methods are not directly comparable, given the circularity in the datasets. The results are available in the Data S1 and can be used as an upper performance estimate for these methods. In this regard, we note that logistic regression based on PFS3 has a significantly larger AUC (*P* = 1.4 × 10^−3^) than Condel, and that all models except SIFT have a significantly larger AUC than PolyPhen‐2 and CADD, even though these methods have the unfair advantage of circularity on the datasets (see Data S1).

**Table 2:** Performance of classifiers

Classifier^a^	Features^b^	Cutoff	Sensitivity	Specificity	Accuracy	PPV	NPV	MCC	AUC	*P*‐value^c^	Significant^d^
PFS3 LR	PON‐P2, GO.BP, PROVEAN	0.525^e^	0.885	0.884	0.885	0.905	0.860	0.767	0.946	4.2 × 10^−3^	Yes
PON‐P2	–	0.5^f^	0.795	0.931	0.855	0.935	0.783	0.722	0.940	1.3 × 10^−6^	Yes
PFS2 LR	GO.BP, PROVEAN, SIFT, GERP, disordered.region	0.557^e^	0.854	0.851	0.853	0.878	0.823	0.703	0.915	1.6 × 10^−13^	Yes
PROVEAN	–	2.282^f^	0.826	0.802	0.815	0.840	0.786	0.627	0.879	1.4 × 10^−2^	Yes
PFS1 LR	GO.BP, evolutionary.rate, disordered.region	0.577^e^	0.795	0.802	0.798	0.834	0.757	0.594	0.861	3.5 × 10^−1^	No
SIFT	–	0.05^f^	0.798	0.776	0.788	0.817	0.754	0.572	0.859	–	–

PFS, predictive feature set; LR, logistic regression; PPV, positive predictive value; NPV, negative predictive value; MCC, Matthew's correlation coefficient; AUC, area under the curve.

a Ranked by AUC.

b Constituting features for the predictive feature sets.

c Bootstrap test for difference in AUC to next ranking classifier.

d Whether the difference in AUC is significant at a 0.05 threshold.

e Cutoff that maximizes the MCC.

f Cutoff as proposed by the program developers.

We further compared the sensitivities of the different approaches at the specificity reported for PON‐P2 by its developers (0.86) to better reproduce results in a real application scenario. At the given specificity level, the sensitivities obtained by the different methods in general agree with their AUC ranking (see Fig. 6B). Finally, we computed precision–recall curves for all methods, which show a similar performance as the ROC curves (see Fig. S5). The distribution of the logistic regression predictions on PFS1, PFS2, and PFS3 are shown in Fig. S6.

It is noteworthy that the best and the third best performing approaches combine results of recent prediction methods with the GO.BP feature, indicating that it is still possible to improve performance of current methods. The second best performing method, PON‐P2 incorporates a GO feature which measures the over‐representation of a gene's GO term in genes of pathogenic variants. Hence, multifunctionality, as reflected by GO annotations, appears to be an important indicator for the pathogenicity of a variant in that gene.

### Systematic miss‐classification of SAPs

**Figure 7:**
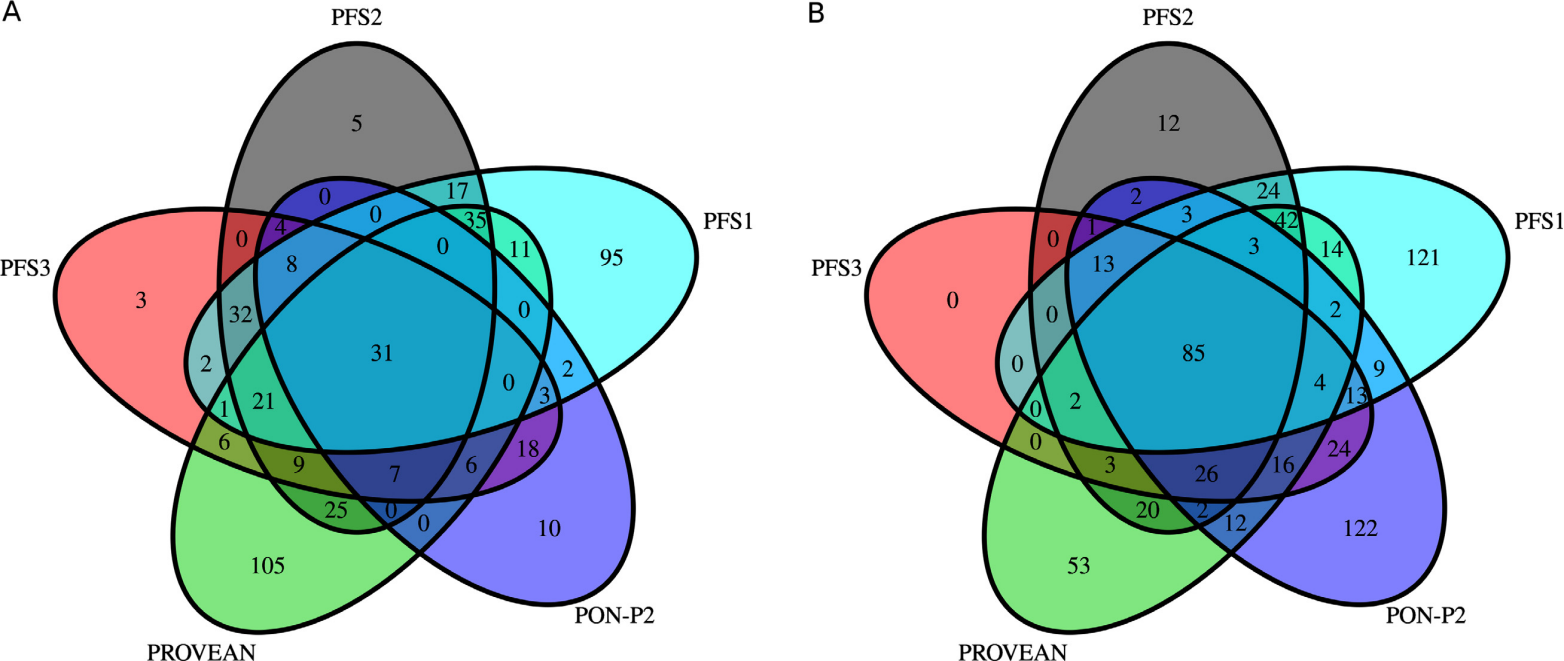
Venn diagram showing the overlap of false predictions on the *validation* set by PON‐P2, PROVEAN, and the logistic regression models trained on the predictive feature sets (PFS) 1, 2, and 3. (A) False positives (single amino acid polymorphisms falsely predicted as pathogenic). (B) False negatives (single amino acid polymorphisms falsely predicted as benign).

An important question is whether there exists a group of SAPs where all methods tend to fail in making correct predictions. To address this issue, we selected the representative candidates PON‐P2, PROVEAN, and the logistic regression models trained on the PFSs. We used the cutoffs listed in Table 2 for all methods. PFS1, PFS2, and PFS3 were trained on the *training* set and predictions were computed for the *validation* set. Values for PON‐P2 and PROVEAN were taken from the *validation* set. We determined which SAPs were falsely predicted as pathogenic (false positives) and falsely predicted as benign (false negatives, FN). Between 11% (PFS3) and 20% (PFS1) of pathogenic and 7% (PON‐P2) and 20% (PROVEAN) of benign SAPs were incorrectly predicted by an individual method, while only 5% of pathogenic and 2% of benign SAPs (85 and 31, respectively) were incorrectly predicted by all five methods (see Fig. 7). We label SAPs falsely classified by all five methods “difficult SAPs”. That is, about 19% of a method's false positives and about 32% of a method's FNs were difficult SAPs. Analysis of the features constituting PFS1, PFS2, and PFS3 showed that their distribution in the false positive and FN SAPs is similar to their distribution in the true positive and true negative SAPs, respectively. Further, none of the remaining features can be used to distinguish false from true predictions (see Fig. S7). However, while the mean 1000 Genomes phase 3 allele frequency was 0.001 for both the true positives in the *validation* set and the difficult FN SAPs, the mean allele frequency in the difficult false positive SAPs was 0.068, which was lower than the mean allele frequency of 0.165 of the true negative SAPs in the *validation* set. Next, we checked if there were genes that had a higher number of difficult SAPs than expected given the total number of variants in this gene and the error rate of 5% and 2% for pathogenic and benign SAPs, respectively. Twenty genes had more than one difficult SAP (see Table S4), six genes even had more than three difficult FN SAPs. Of these, five genes had a frequency of FN SAPs higher than 5% given the total number of SAPs for this gene in the *validation* set: Five of seven SAPs in *RAI1* (OMIM:607642) are FN with “Smith–Magenis syndrome” HGMD annotations. Four of 48 SAPs in *MSH6* (OMIM:600678) are FN with “colorectal cancer” HGMD annotations. Four of 30 SAPs in *PEX6* (OMIM:601498) are FN with “Peroxisome biogenesis disorder/Zellweger syndrome” HGMD annotations. Four of four SAPs in *ROM1* (OMIM:180721) are FN with “Retinitis pigmentosa/macular dystrophy modifier” HGMD annotations. Four of seven SAPs in *CRELD1* (OMIM:607170) are FN with “Cardiac atrioventricular septal defect” HGMD annotations. Possibly, these genes or diseases possess particular characteristics different from other disease genes or disease phenotypes that cause the misclassification of the pathogenic SAPs in these genes. The set of difficult cases (without SAPs from HGMD) is available as Table S5.

## Discussion

In this study, we have assessed novel features and existing methods for predicting the pathogenicity of SAPs. We have found the combination of PON‐P2, GO.BP, and PROVEAN to perform best on our *validation* set. The evaluation of this model is not biased by circularity in our data. However, PON‐P2 is correlated with both GO.BP and PROVEAN, which might lead to an overoptimistic evaluation of the PFS 3. On the other hand, the feature selection with both logistic regression and random forests resulted in the same set of features. Given that the random forest implementation used in this study accurately evaluates feature importance even in the presence of correlated features, this increases our confidence in the results. The features selected by the decision trees are similar to the features selected with random forests and logistic regression in the forward selection. These results suggest that the classification rules determined by the decision trees provide meaningful interpretations of the characteristics of pathogenic and benign SAPs. We conclude that by combining the GO.BP gene feature with existing methods, the prediction performance can be further improved.

The gene‐based feature GO.BP has been included in all PFSs indicating that it provides a substantial contribution to pathogenicity prediction. As GO.BP is the number of “BP” Gene Ontology (GO) annotations, it reflects a measure of gene multifunctionality. We found that a gene's number of GO BP annotations was not biased by whether or not pathogenic variants had been reported in the gene, as the knowledge of pathogenic variants in genes did not lead to a significantly higher increase in the genes’ number of annotations from 2008 to 2013. Genes unknown to actually harbor pathogenic variants further had a significantly higher increase in annotations from 2008 to 2013 than neutral genes, which indicates that gene multifunctionality, as captured by the GO.BP feature, is a true characteristic of disease genes. On the other hand, the GO.BP feature might suffer from an additional bias that is independent of pathogenic variants, as genes might also be more intensively studied if they are known to be involved in disease pathways or interact with disease genes, leading likewise to a higher number of GO annotations compared to non‐disease genes. Consequently, it is possible that a model using this feature will perform worse on pathogenic SAPs in genes that have not yet been associated with disease, but this effect will dissolve, as with the advance of next‐generation sequencing most disease genes are being identified.

Notably, GO.BP was the only gene‐based feature that improved prediction performance. However, other gene‐based features might benefit from improved underlying data. In the case of the network features (degree, centrality, and betweenness), which have been successfully used for pathogenicity prediction before (Huang et al. 2012; Yates et al. 2014), a more comprehensive and reliable human interaction network might improve the prediction performance. The gene expression feature, which measures the fraction of tissues in which the gene was expressed, is likely not sensitive enough to capture the disease and tissue‐specific effects. With the increase in next‐generation sequencing efforts, higher quality transcriptome data will likely be available in the near future.

We have identified a set of difficult SAPs, where PON‐P2, PROVEAN, and the logistic regression models based on PFS1, PFS2, and PFS3 all fail in their prediction. We acknowledge the possibility that the predictors are actually correct, and that these SAPs were initially assigned to the wrong set in the design of the datasets. That is, they might represent erroneous pathogenic entries in HGMD or ClinVar or might be pathogenic SAPs despite a high allele frequency in the 1000 Genomes project due to low penetrance. Nevertheless, with the chosen cutoff, 83% of a method's false positives and 87% of a method's FNs were correctly predicted by at least one other method. This is an interesting finding, given that our PFSs already combine features and existing methods, especially PON‐P2 and PROVEAN. We conclude that each method has individual strengths and weaknesses and that, despite the employment of consensus predictors, considering multiple methods yields the highest accuracy.

HGMD and ClinVar are extremely valuable resources in the investigation of the genetic basis of disease, nevertheless the lack of penetrance data and uniform criteria for defining pathogenic variants are considerable limitations, though there are some efforts to address the latter point (Richards et al. 2015). As a result, the derived sets of pathogenic variants are very heterogeneous, which negatively affects the analysis and further progress in the field.

For practicability reasons most previous studies, including this one, have focused on the effects of SAPs. Consequently, many prediction scores (e.g., SIFT, PolyPhen‐2, and Condel) are not applicable to indels or multi‐nucleotide polymorphisms. However, two of our top features, GO.BP and PROVEAN are defined for any small genetic variation and are promising candidate features for improved prediction programs.

## Conflict of Interest

None declared.

## Supporting information


**Data S1.** Methods and results.
**Figure S1.** Venn diagram showing the number and overlap of the SAPs’ genes. Benign = unique number of genes of the benign SAPs; pathogenic = unique number of genes of the pathogenic SAPs. (A) *training* data set. (B) *validation* data set.
**Figure S2.** Overlap of the training and validation datasets with ExoVar, HumVar, SwissVar, and VariBench. (A) Overlap of the datasets for the pathogenic SAPs. (B) Overlap of the datasets for the benign SAPs.
**Figure S3.** Receiver operating characteristic (ROC) curves of the logistic regression models and prediction scores on the *validation* dataset for alternative feature sets to PFS2. (A) Full ROC curve. The vertical dashed line at 0.86 corresponds to the specificity of PON‐P2 as estimated by the authors. (B) Same data as in (A), zoomed into the region where the lines of the ROC curve intersect the specificity threshold of 0.86.
**Figure S4.** Distribution of values in pathogenic (P) and benign (B) variants for the 21 features not in PFS1, PFS2, or PFS3 on the *training* set and the trained prediction scores PolyPhen‐2, Condel, and CADD. (A) PolyPhen‐2. (B) Condel. (C) CADD. (D) Grantham. (E) accessibility. (F) secondary.structure.3. (G) secondary.structure.8. (H) PfamA. (I) AAindex.polarity. (J) AAindex.hydropathy. (K) AAindex.volume. (L) AAindex.composition. (M) AAindex.net.charge. (N) protein.age. (O) paralog.nr. (P) paralog.id. (Q) mouse.orth.nr. (R) mouse.orth.id. (S) expression. (T) degree. (U) centrality. (V) betweenness. (W) gene.length. (X) protein.length.
**Figure S5.** Precision–Recall curves for all methods accessed in the main analysis.
**Figure S6.** Prediction of logistic regression models, trained on the *training* set and predicted on the *validation* set. P = true disease class is pathogenic, B = true disease class is benign. The dotted red line corresponds to the threshold at the maximal Matthew's correlation coefficient to classify SAPs as pathogenic or benign. (A) PFS1 (class ~ GO.BP + evolutionary.rate + disordered.region); threshold = 0.577. (B) PFS2 (class ~ GO.BP + PROVEAN + SIFT + GERP + disordered.region); threshold = 0.557. (C) PFS3 (class ~ PON‐P2 + GO.BP + PROVEAN); threshold = 0.525.
**Figure S7.** Distribution of feature values (and PolyPhen‐2, Condel, CADD) on subsets of the *validation* set based on the prediction overlap of PFS1, PFS2, PFS3, PON‐P2, and PROVEAN. SAP, single amino acid polymorphism; FP, SAPs falsely predicted pathogenic by all five methods; FN, SAPs falsely predicted benign by all five methods; TP, SAPs correctly predicted pathogenic by all five methods; TN, SAPs correctly predicted benign by all five methods. (A) PolyPhen‐2. (B) Condel. (C) CADD. (D) PON‐P2. (E) SIFT. (F) PROVEAN. (G) Grantham. (H) GERP. (I) evolutionary.rate. (J) disordered.region. (K) accessibility. (L) secondary.structure.3. (M) secondary.structure.8. (N) PfamA. (O) AAindex.polarity. (P) AAindex.hydropathy. (Q) AAindex.volume. (R) AAindex.composition. (S) AAindex.net.charge. (T) protein.age. (U) paralog.nr. (V) paralog.id. (W) mouse.orth.nr. (X) mouse.orth.id. (Y) GO.BP. (Z) expression. A2: degree. B2: centrality. C2: betweenness. D2: gene.length. E2: protein.length.
**Figure S8.** Feature selection using all features as input, including the trained prediction scores Condel, PolyPhen‐2, and CADD that suffer from circularity on the *training* and *validation* set. (A) Decision tree computed on the *training* set. Each tree node has three rows: the upper row contains the decision made at this node with 0 = benign and 1 = pathogenic; the second row shows the fraction of single amino acid polymorphisms (SAPs) classified at this node as benign (left) and pathogenic (right); the third row shows the percentage of all input SAPs that are classified at this node. Starting from the root node, at each node, the left child is traversed if the condition evaluates to true and the right child is traversed if the condition evaluates to false. (B) Cross‐validation (CV) test error of stepwise forward selection with random forest and linear regression. Points correspond to the mean test error from all CV iterations with error bars. The label corresponds to the feature that was added in this step and the number below indicates the percentage of CV iterations in which this feature was selected. Labels are printed above and below the points for random forest and linear regression, respectively. The vertical dashed line shows the cutoff for feature selection. Only the first 10 steps of the forward selection are shown.
**Figure S9.** Receiver operating characteristic (ROC) curves showing specificity versus sensitivity of the logistic regression models including predictive feature set 4 (PFS4) and prediction scores at different thresholds on the *validation* set. In the legend, models are ordered according to their AUC values. Some lines are dotted to improve visibility. (A) Full ROC curve. The vertical dashed line at 0.86 corresponds to the specificity of PON‐P2 as estimated by the developers. (B) Same data as in (A), zoomed into the region where the lines of the ROC curve intersect the specificity threshold of 0.86.
**Figure S10**. Precision–Recall curves for all methods accessed in this study, as well as Condel, PolyPhen‐2, and CADD.
**Table S1.** Number of pathogenic and benign single amino acid polymorphisms employed in the feature selection with decision trees, logistic regression, and random forests in the *training* set.
**Table S2.** Results of the silhouette width analysis on the features clustered based on their absolute correlation. Clusters were ordered according to their mean silhouette width values.
**Table S3.** Classifier performance including the trained prediction scores PolyPhen‐2, Condel, and CADD that suffer from circularity on the *training* and *validation* set. SAPs containing NA values in any features constituting the PFS or the comparison scores were removed from the training and validation sets.
**Table S4.** Genes of the *validation* set that have at least two “difficult SAPs”. FN, false negative; FP, false positive.

**Table S5.** “Difficult SAPs” (without SAPs from HGMD).
