# Supplementary material for: Computational assessment of feature combinations for pathogenic variant prediction
Source: Mol Genet Genomic Med. 2016 Mar 14;4(4):431–46. doi: 10.1002/mgg3.214 (PMC4947862; doi:10.1002/mgg3.214)
Supplement: Supplementary file 1 — Data S1. Methods and results. Figure S1. Venn diagram showing the number and overlap of the SAPs’ genes. Benign = unique number of genes of the benign SAPs; pathogenic = unique number of genes of the pathogenic SAPs. (A) training data set. (B) validation data set. Figure S2. Overlap of the training and validation datasets with ExoVar, HumVar, SwissVar, and VariBench. (A) Overlap of the datasets for the pathogenic SAPs. (B) Overlap of the datasets for the benign SAPs. Figure S3. Receiver operating characteristic (ROC) curves of the logistic regression models and prediction scores on the validation dataset for alternative feature sets to PFS2. (A) Full ROC curve. The vertical dashed line at 0.86 corresponds to the specificity of PON‐P2 as estimated by the authors. (B) Same data as in (A), zoomed into the region where the lines of the ROC curve intersect the specificity threshold of 0.86. Figure S4. Distribution of values in pathogenic (P) and benign (B) variants for the 21 features not in PFS1, PFS2, or PFS3 on the training set and the trained prediction scores PolyPhen‐2, Condel, and CADD. (A) PolyPhen‐2. (B) Condel. (C) CADD. (D) Grantham. (E) accessibility. (F) secondary.structure.3. (G) secondary.structure.8. (H) PfamA. (I) AAindex.polarity. (J) AAindex.hydropathy. (K) AAindex.volume. (L) AAindex.composition. (M) AAindex.net.charge. (N) protein.age. (O) paralog.nr. (P) paralog.id. (Q) mouse.orth.nr. (R) mouse.orth.id. (S) expression. (T) degree. (U) centrality. (V) betweenness. (W) gene.length. (X) protein.length. Figure S5. Precision–Recall curves for all methods accessed in the main analysis. Figure S6. Prediction of logistic regression models, trained on the training set and predicted on the validation set. P = true disease class is pathogenic, B = true disease class is benign. The dotted red line corresponds to the threshold at the maximal Matthew's correlation coefficient to classify SAPs as pathogenic or benign. (A) PFS1 (class ~ GO.BP + evolutionary.rate + [file MGG3-4-431-s001.docx]

**Supplementary Information**

**Supplementary Methods**

**Dintor [Weichenberger et al., 2015] commands to create GO.BP feature**

The file uniprot.txt is a text file that contains the UniProt identifiers of all genes in the *training* and *validation* set, one per line.

This is the command to generate the GO.BP feature on the GO 2014 data (the feature used in the main analysis). Output is printed to stdout.

$ python Dintor/src/python/cmd/GOAnnotator.py --data-version v-201411 --in uniprot --limit-term-category BP -c 1 --ic --from-file uniprot.txt --remove-children --thres 2 -H --term-id --term-name --term-category --evidence

This is the command to generate the GO.BP feature on the GO 2008 data (used for the assessment of the GO bias). Output is printed to stdout.

$ python Dintor/src/python/cmd/GOAnnotator.py --data-version v-200801 --in uniprot --limit-term-category BP -c 1 --ic --from-file uniprot.txt --remove-children --thres 2 -H --term-id --term-name --term-category --evidence

**Handling of Missing Data**

There were 14,033 pathogenic and 15,574 benign single amino acid polymorphisms (SAPs) in the *training* and 2,085 pathogenic and 2,351 benign SAPs in the *validation* set. However, not all of these SAPs had values defined at all 28 features. To maximize the number of variants for training and testing, different strategies were applied to handle NA values. For the correlation analysis on the *training* data set, feature correlation was computed on all SAPs that were pairwise complete for each pair of features. For the feature selection, we used a three step approach considering different types of features in each step. We removed any SAPs from the *training* set that had a NA value in any of the features that were considered at this step. Supp. Table S1 lists the number of SAPs employed in each step. For the training and validation of the three models build on the three predictive feature sets (PFS) we removed any SAP that had an NA value in any of the features of the PFSs or the comparison scores (for PFS4 in Supp. Results also PolyPhen-2, and CADD). This selection resulted in 10,655 pathogenic and 8,871 benign SAPs in the *training* and in 1,632 pathogenic and 1,300 benign SAPs in the *validation* set.

**Assessing the overlap of the *training* and *validation* sets with ExoVar, HumVar, SwissVar, and VariBench**

To determine the overlap of the SAPs in the training and validation set with the variants in the benchmarking datasets ExoVar, HumVar, SwissVar, and VariBench, the variants of all datasets were converted to the Dintor [Weichenberger at al., 2015] genomic coordinate format. For SAPs, the genomic coordinate is simply represented as [genome build]:[chromosome]:[position]:[reference allele]:[alternative allele]. The ExoVar dataset was provided in such a format, so no conversion was necessary. In the HumVar and SwissVar datasets, the UniProt gene id, amino acid position, reference amino acid, and mutated amino acid was given for each variant. To convert these variants, the PICMI online server [Le Pera et al., 2010] was used. Conversions were successful in 80% of the HumVar and SwissVar variants. For the VariBench pathogenic variants complete HGVS protein IDs including the reference sequence IDs were given. These HGVS ids were translated to vcf format with the variant effect predictor (VEP) script version 75 from Ensembl with the following command. HGVS.txt is a text file with one HGVS id per line.

$ perl variant_effect_predictor.pl --database --refseq --convert vcf --cache --cache_version 83 -i HGVS.txt > HGVS.vcf

The derived vcf file was translated to genomic coordinate format with the Dintor tool VCF2Dint.py.

$ python Dintor/src/python/cmd/VCF2Dint.py --build GRCh37 HGVS.vcf

For the VariBench neutral variants, dbSNP ids from version 131 were given for all entries. However, the oldest available dbSNP version was 137. The dbSNP 137 file in vcf format was subset to include only the VariBench variants, which was successful for 91% of the variants. The VCF file was translated to genomic coordinate format with the Dintor tool VCF2Dint.py.

**Supplementary Results**

**Feature Selection and Model Evaluation including the biased scores PolyPhen-2, Condel, and CADD**

We repeated the analysis as described in the main document including PolyPhen-2, Condel, and CADD. Even though these trained prediction scores suffer from circularity on our *training* and *validation* set, they provide an upper boundary for their performance. Further if an unbiased predictor performs better than these scores or a method including them, one can conclude that the unbiased predictor truly performs better.

As described in the Methods section of the main document, we first computed a decision tree on the *training* set including all features and Condel, PolyPhen-2, and CADD. The features included in the tree were Condel and PON-P2 (see Supp. Figure S8A). Next we performed the cross-validation forward selection using random forests and logistic regression. Both approaches identified Condel, PON-P2, and GO.BP as the most predictive features (see Supp. Figure S8B). These three features were selected to form the predictive feature set 4 (PFS4).

Validation of all methods on the *validation* set showed that PFS4 outperformed all other methods (see Supp. Figures S9 and S10, and Supp. Table S3). However, with the inclusion of Condel, PFS4 suffers from circularity on our datasets and its good performance is likely at least partially due to this bias.

| **Features** | **Pathogenic** | **Benign** |
| --- | --- | --- |
| Raw features | 10,304 | 7,319 |
| Raw features and rule prediction scores | 9,968 | 6,725 |
| All features | 9,833 | 6,471 |
| All features and PolyPhen-2, Condel, CADD | 9,823 | 4,933 |

**Supp. Table S1.** Number of pathogenic and benign single amino acid polymorphisms employed in the feature selection with decision trees, logistic regression, and random forests in the *training* set.

| **Cluster number** | **Number of features** | **Features** | **Mean silhouette value** |
| --- | --- | --- | --- |
| 1 | 2 | AAindex.polarity, AAindex.hydropathy | 0.69 |
| 2 | 3 | degree, betweeness, GO.BP | 0.55 |
| 3 | 2 | mouse.orth.id, mouse.orth.nr | 0.54 |
| 4 | 2 | paralogs.id, paralogs.nr | 0.49 |
| 5 | 2 | AAindex.volume, AAindex.composition | 0.37 |
| 6 | 8 | PON-P2, PROVEAN, SIFT, GERP, Grantham, disordered.region, evolutionary.rate, accessibility | 0.28 |
| 7 | 2 | gene.length, protein.length | 0.21 |
| 8 | 2 | protein.age, expression | 0.08 |
| 9 | 1 | centrality | 0.00 |
| 10 | 1 | AAindex.net.charge | 0.00 |

**Supp Table S2.** Results of the silhouette width analysis on the features clustered based on their absolute correlation. Clusters were ordered according to their mean silhouette width values.

| Classifier^a^ | Features^b^ | Cutoff | Sensitivity^e^ | Specificity | Accuracy | PPV | NPV | MCC | AUC | p-value^c^ | Significant^d^ |
| --- | --- | --- | --- | --- | --- | --- | --- | --- | --- | --- | --- |
| PFS4 LR*** | Condel, PON-P2, GO.BP | 0.488* | 0.929 | 0.869 | 0.906 | 0.821 | 0.882 | 0.800 | 0.941 | 5.1x10^-10^ | yes |
| PFS3 LR | PON-P2, GO.BP, PROVEAN | 0.523* | 0.903 | 0.868 | 0.890 | 0.918 | 0.844 | 0.767 | 0.946 | 3.8x10^-3^ | yes |
| PON-P2 | - | 0.5** | 0.795 | 0.933 | 0.847 | 0.951 | 0.735 | 0.706 | 0.939 | 8.2x10^-2^ | no |
| Condel*** | - | 0.522** | 0.844 | 0.873 | 0.855 | 0.916 | 0.773 | 0.703 | 0.931 | 6.6x10^-3^ | yes |
| PFS2 LR | GO.BP, PROVEAN, SIFT, GERP, disordered.region | 0.606* | 0.859 | 0.855 | 0.858 | 0.907 | 0.787 | 0.704 | 0.916 | 1.1x10^-9^ | yes |
| PROVEAN | - | 2.282** | 0.826 | 0.819 | 0.823 | 0.882 | 0.741 | 0.634 | 0.883 | 1.1x10^-2^ | yes |
| PFS1 LR | GO.BP, evolutionary.rate, disordered.region | 0.587* | 0.838 | 0.757 | 0.807 | 0.850 | 0.740 | 0.592 | 0.863 | 3.4x10^-1^ | no |
| SIFT | - | 0.05** | 0.798 | 0.782 | 0.792 | 0.857 | 0.702 | 0.569 | 0.859 | 2.0x10^-1^ | no |
| CADD*** | - | 15.87* | 0.908 | 0.646 | 0.809 | 0.808 | 0.811 | 0.585 | 0.852 | 4.2x10^-18^ | yes |
| PolyPhen-2*** | - | 0.85** | 0.591 | 0.874 | 0.698 | 0.885 | 0.566 | 0.458 | 0.770 | - | - |

PFS: predictive feature set; LR: logistic regression; PPV: positive predictive value; NPV: negative predictive value; MCC: Matthew's correlation coefficient; AUC: area under the curve

a Ranked by AUC; PFS = predictive feature set; LR = logistic regression

b Constituting features for the predictive feature sets
c Bootstrap test for difference in AUC to next ranking classifier

d Whether the difference in AUC (b) is significant at a 0.05 threshold

* Cutoff that maximizes the MCC.

** Cutoff as proposed by the program developers.

*** Predictors suffer from circularity on the *training* and *validation* set.

**Supp. Table S3:** Classifier performance including the trained prediction scores PolyPhen-2, Condel, and CADD that suffer from circularity on the *training* and *validation* set. SAPs containing NA values in any features constituting the PFS or the comparison scores were removed from the training and validation sets.

| **Type** | **Number of difficult SAPs** | **Total number of SAPs** | **Fraction difficult SAPs** | **HGNC gene name** | **HGMD disease term** |
| --- | --- | --- | --- | --- | --- |
| FN | 15 | 394 | 0.04* | MYH7 | hypertrophic or dilated Cardiomyopathy, Myopathypathy |
| FN | 5 | 7 | 0.71 | RAI1 | Smith-Magenis syndrome |
| FN | 4 | 48 | 0.08 | MSH6 | Colorectal or endometrial cancer (early onset) |
| FN | 4 | 30 | 0.13 | PEX6 | Peroxisome biogenesis disorder, Zellweger syndrome |
| FN | 4 | 4 | 1.00 | ROM1 | Retinitis pigmentosa, PRPH2-associated macular dystrophy modifier |
| FN | 4 | 7 | 0.57 | CRELD1 | Cardiac atrioventricular septal defect |
| FN | 3 | 4 | 0.75 | NEBL | Cardiomyopathy, dilated & endocardial fibroelastosis |
| FN | 3 | 5 | 0.60 | GNPTG | Mucolipidosis III, Stuttering |
| FN | 3 | 5 | 0.60 | WDR11 | Hypogonadotropic hypogonadism |
| FN | 3 | 57 | 0.05* | F13A1 | Factor XIII deficiency (A type II) |
| FN | 3 | 13 | 0.23 | ROBO3 | horizontal gaze palsy, with progressive scoliosis |
| FN | 3 | 5 | 0.60 | SCNN1G | Bronchiectasis, Hypertension |
| FN | 2 | 5 | 0.40 | GRIP1 | Autism |
| FN | 2 | 10 | 0.20 | LIPG | Reduced lipase activity, Low HDL cholesterol |
| FN | 2 | 9 | 0.22 | MTNR1B | type 2 Diabetes |
| FN | 2 | 34 | 0.06 | PCNT | Intellectual disability |
| FN | 2 | 3 | 0.67 | SUGCT | Complex I deficiency |
| FP | 2 | 105 | 0.02* | COMP | NA |
| FP | 2 | 7 | 0.29 | PKD2L1 | NA |
| FP | 2 | 4 | 0.50 | TNIP2 | NA |

* This proportion of difficult SAPs is less than or equal to the proportion expected per gene, given the difficult SAP error rate of 5% and 2% for pathogenic and benign SAPs, respectively.

**Supp. Table S4.** Genes of the *validation* set that have at least two “difficult SAPs”. FN = false negative, FP = false positive.


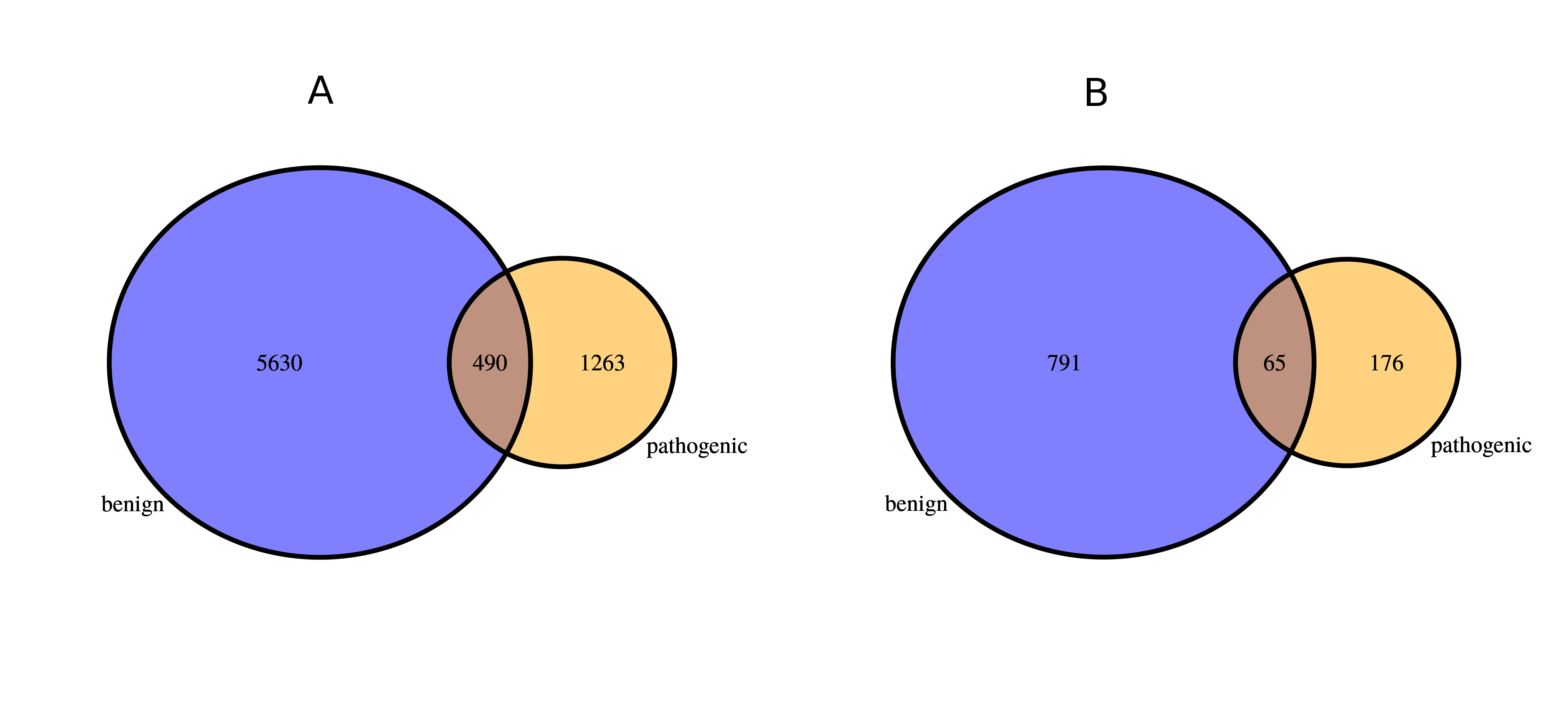


**Supp. Figure S1:** Venn diagram showing the number and overlap of the SAPs' genes. Benign = Unique number of genes of the benign SAPs; pathogenic = Unique number of genes of the pathogenic SAPs. **A:** *training* data set. **B:** *validation* data set.


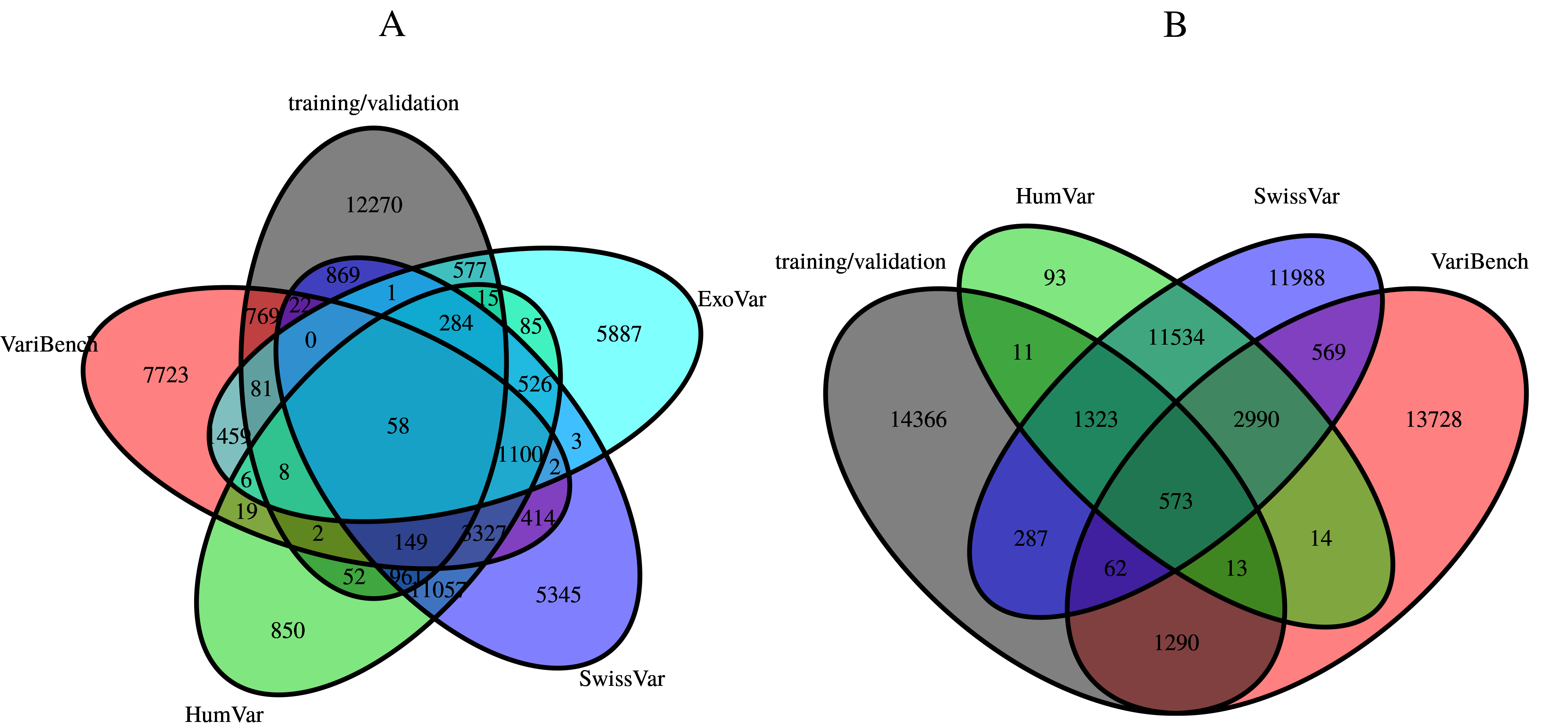


**Supp. Figure S2.** Overlap of the training and validation datasets with ExoVar, HumVar, SwissVar, and VariBench. **A**: Overlap of the datasets for the pathogenic SAPs. **B**: Overlap of the datasets for the benign SAPs.


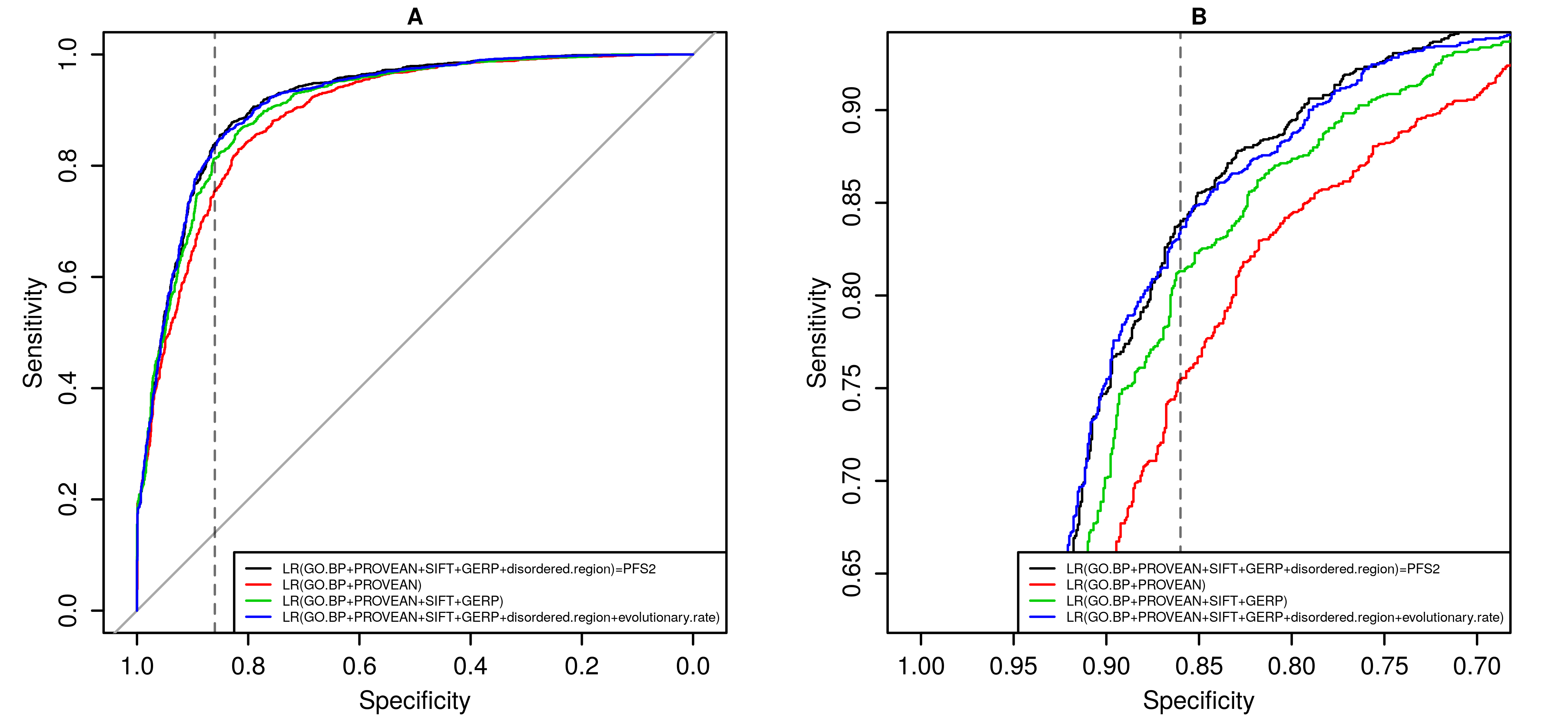


**Supp. Figure S3.** Receiver operating characteristic (ROC) curves of the logistic regression models and prediction scores on the *validation* dataset for alternative feature sets to PFS2. **A**: Full ROC curve. The vertical dashed line at 0.86 corresponds to the specificity of PON-P2 as estimated by the authors. **B**: Same data as in **A**, zoomed into the region where the lines of the ROC curve intersect the specificity threshold of 0.86.

**Supp. Figure S4** Distribution of values in pathogenic (P) and benign (B) variants for the 21 features not in PFS1, PFS2, or PFS3 on the *training* set and the trained prediction scores PolyPhen-2, Condel, and CADD. **A**: PolyPhen-2. **B**: Condel. **C**: CADD. **D**: Grantham. **E**: accessibility. **F:** secondary.structure.3. **G**: secondary.structure.8. **H**: PfamA. **I**: AAindex.polarity. **J**: AAindex.hydropathy. **K**: AAindex.volume. **L**: AAindex.composition. **M**: AAindex.net.charge. **N**: protein.age. **O**: paralog.nr. **P**: paralog.id. **Q**: mouse.orth.nr. **R**: mouse.orth.id. **S**: expression. **T**: degree. **U**: centrality. **V**: betweenness. **W**: gene.length. **X**: protein.length.
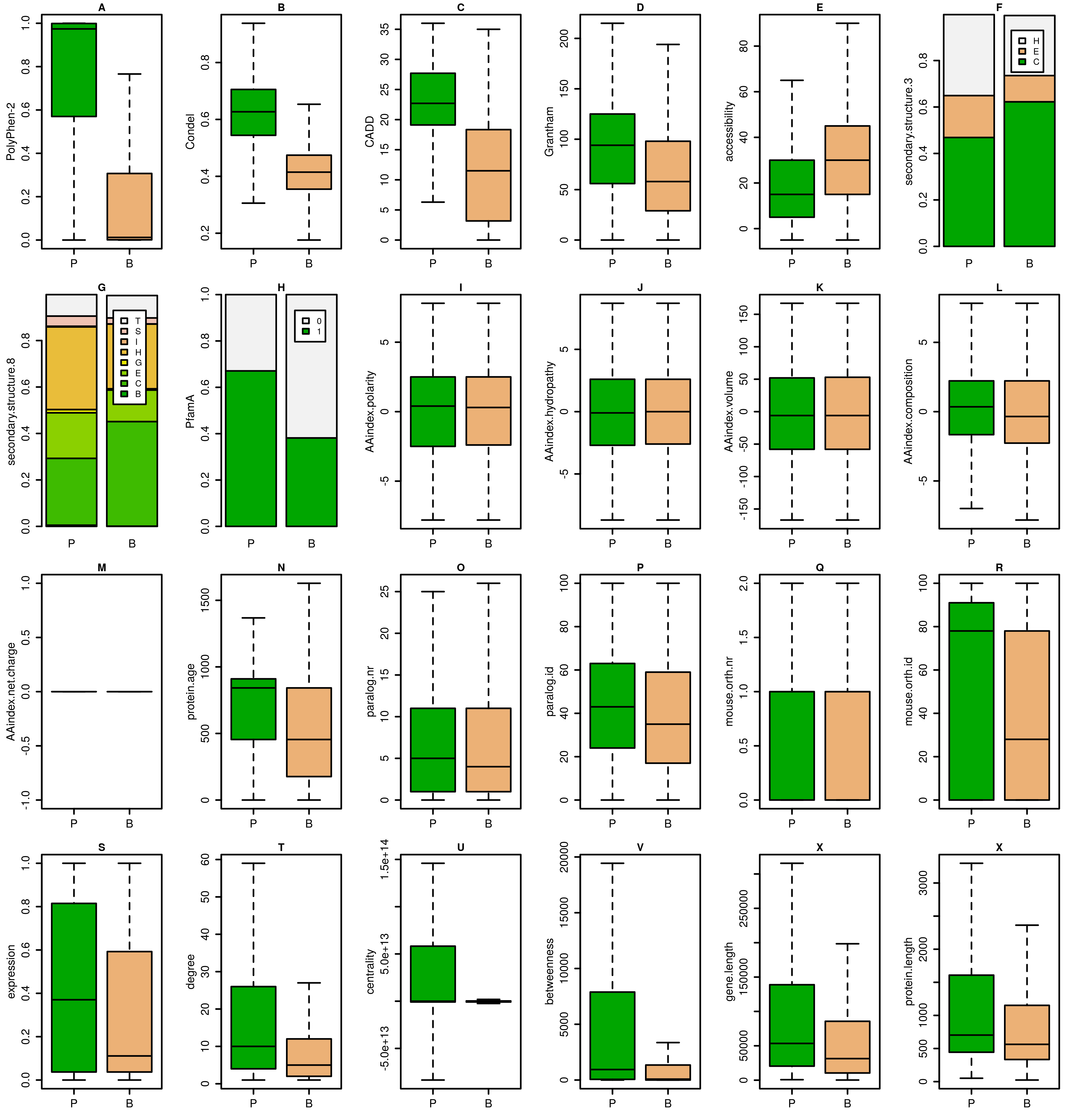


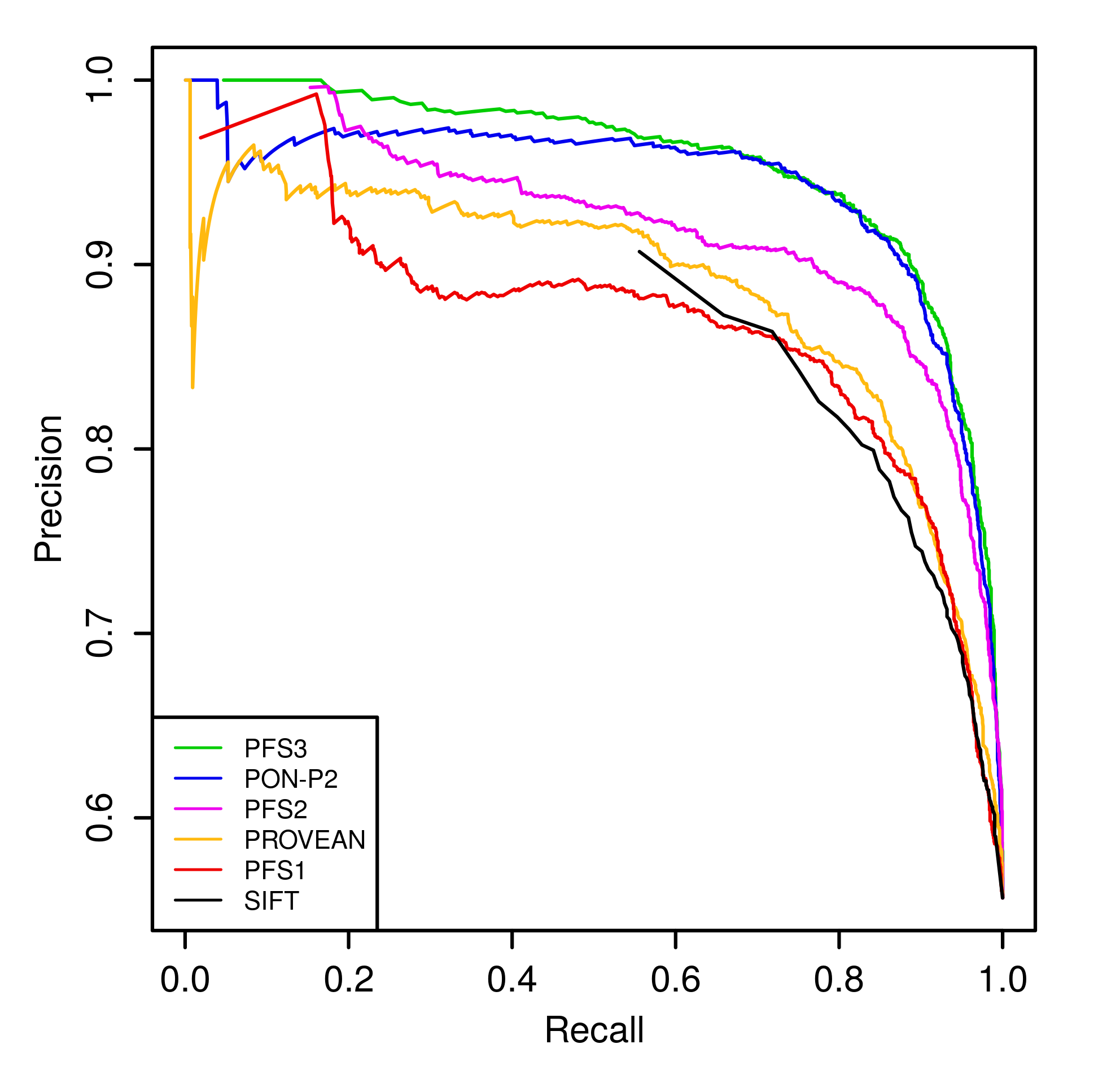


**Supp. Figure S5.** Precision-Recall curves for all methods accessed in the main analysis.


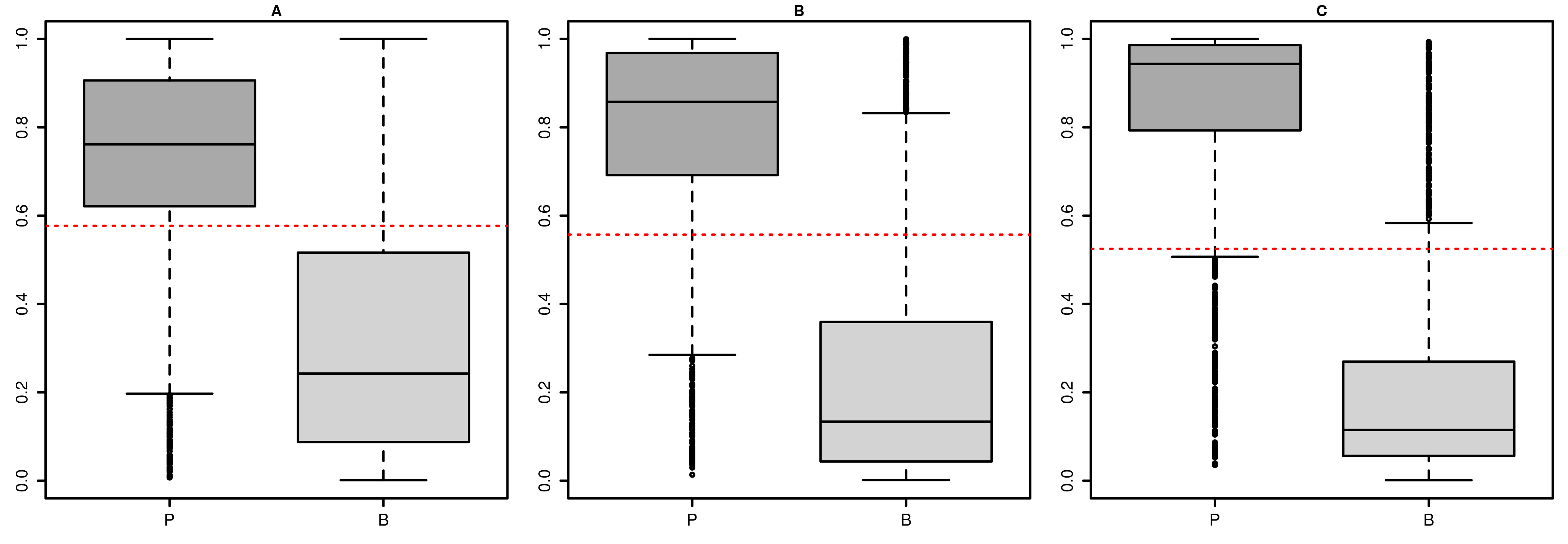


**Supp. Figure S6.** Prediction of logistic regression models, trained on the *training* set and predicted on the *validation* set. P = true disease class is pathogenic, B = true disease class is benign. The dotted red line corresponds to the threshold at the maximal Matthew's correlation coefficient to classify SAPs as pathogenic or benign. **A**: PFS1 (class ~ GO.BP + evolutionary.rate + disordered.region); threshold = 0.577. **B**: PFS2 (class ~ GO.BP + PROVEAN + SIFT + GERP + disordered.region); threshold = 0.557. **C**: PFS3 (class ~ PON-P2+ GO.BP + PROVEAN); threshold = 0.525.


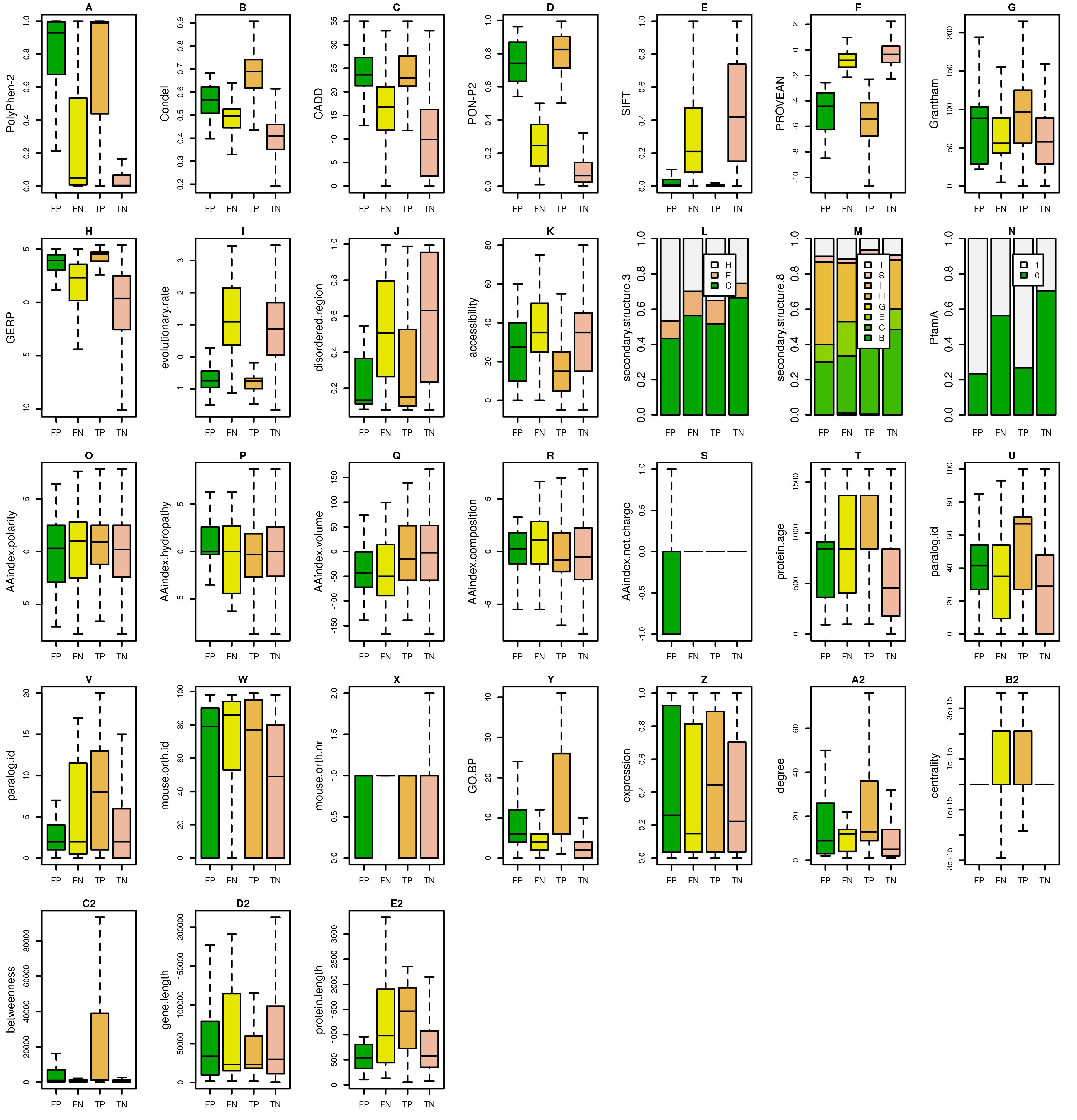


**Supp. Figure S7.** Distribution of feature values (and PolyPhen-2, Condel, CADD) on subsets of the *validation* set based on the prediction overlap of PFS1, PFS2, PFS3 PON-P2, and PROVEAN. SAP = single amino acid polymorphism. FP = SAPs falsely predicted pathogenic by all five methods. FN = SAPs falsely predicted benign by all five methods. TP = SAPs correctly predicted pathogenic by all five methods. TN = SAPs correctly predicted benign by all five methods. **A**: PolyPhen-2. **B**: Condel. **C**: CADD. **D**: PON-P2. **E**: SIFT. **F**: PROVEAN. **G**: Grantham. **H**: GERP. **I**: evolutionary.rate. **J**: disordered.region. **K**: accessibility. **L:** secondary.structure.3. **M**: secondary.structure.8. **N**: PfamA. **O**: AAindex.polarity. **P**: AAindex.hydropathy. **Q**: AAindex.volume. **R**: AAindex.composition. **S**: AAindex.net.charge. **T**: protein.age. **U**: paralog.nr. **V**: paralog.id. **W**: mouse.orth.nr. **X**: mouse.orth.id. **Y**: GO.BP. **Z**: expression. **A2**: degree. B**2**: centrality. **C2**: betweenness. **D2**: gene.length. **E2**: protein.length.


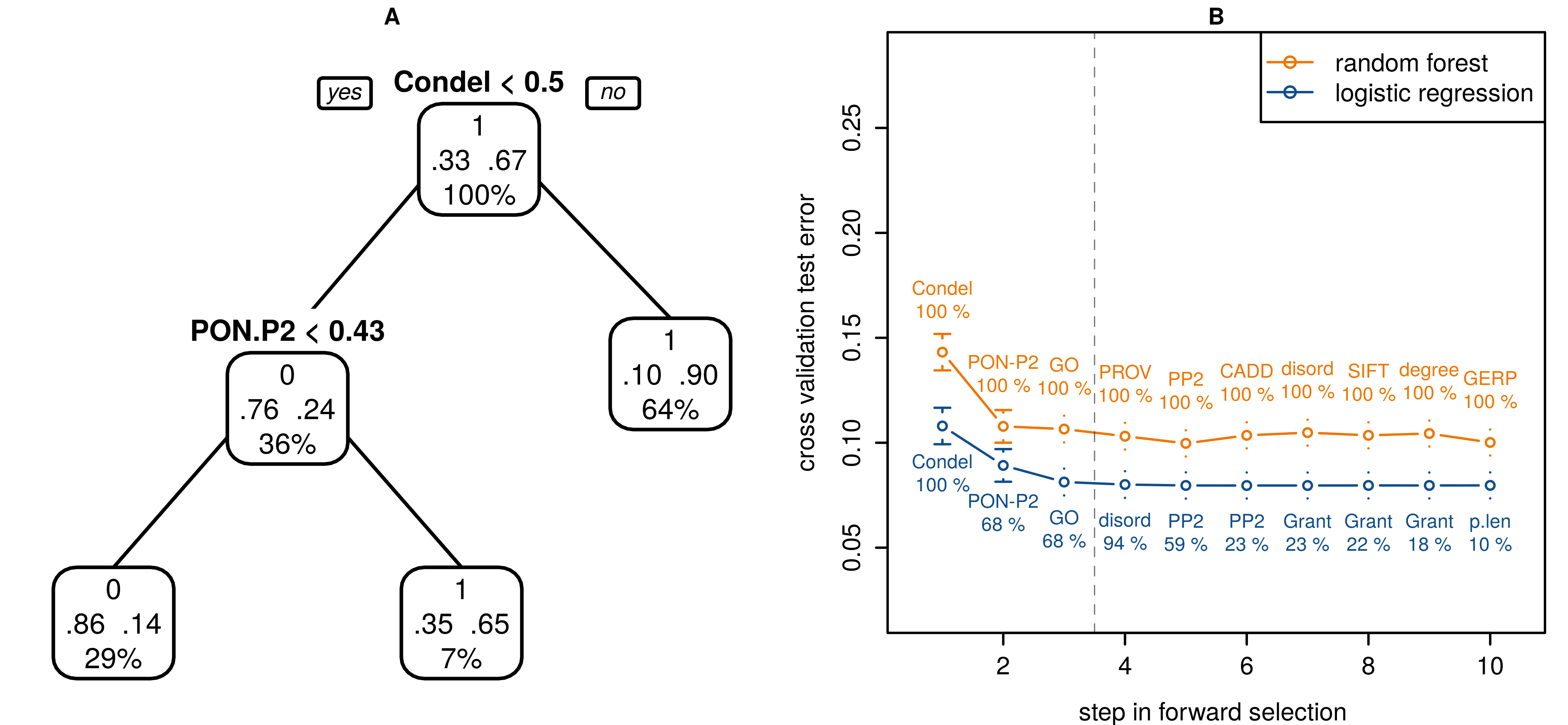


**Supp. Figure S8.** Feature selection using all features as input, including the trained prediction scores Condel, PolyPhen-2, and CADD, that suffer from circularity on the *training* and *validation* set. **A**: Decision tree computed on the *training* set. Each tree node has three rows: the upper row contains the decision made at this node with 0=benign and 1=pathogenic; the second row shows the fraction of single amino acid polymorphisms (SAPs) classified at this node as benign (left) and pathogenic (right); the third row shows the percentage of all input SAPs that are classified at this node. Starting from the root node, at each node the left child is traversed if the condition evaluates to true and the right child is traversed if the condition evaluates to false. **B**: Cross validation (CV) test error of stepwise forward selection with random forest and linear regression. Points correspond to the mean test error from all CV iterations with error bars. The label corresponds to the feature that was added in this step and the number below indicates the percentage of CV iterations in which this feature was selected. Labels are printed above and below the points for random forest and linear regression, respectively. The vertical dashed line shows the cutoff for feature selection. Only the first 10 steps of the forward selection are shown.


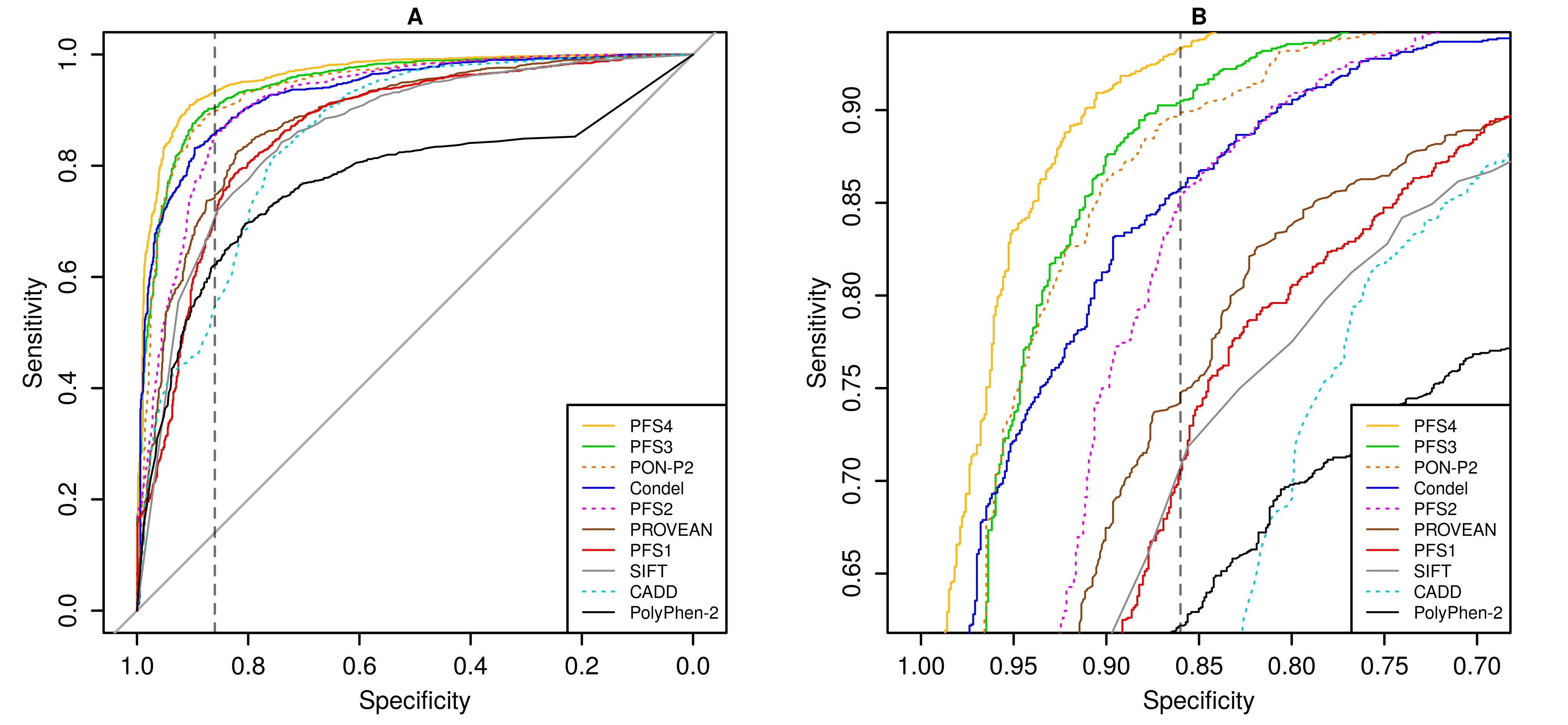


**Supp. Figure S9.**

Receiver operating characteristic (ROC) curves showing specificity versus sensitivity of the logistic regression models including predictive feature set 4 (PFS4) and prediction scores at different thresholds on the *validation* set*.* In the legend models are ordered according to their AUC values. Some lines are dotted to improve visibility. **A**: Full ROC curve. The vertical dashed line at 0.86 corresponds to the specificity of PON-P2 as estimated by the developers. **B**: Same data as in **A**, zoomed into the region where the lines of the ROC curve intersect the specificity threshold of 0.86.


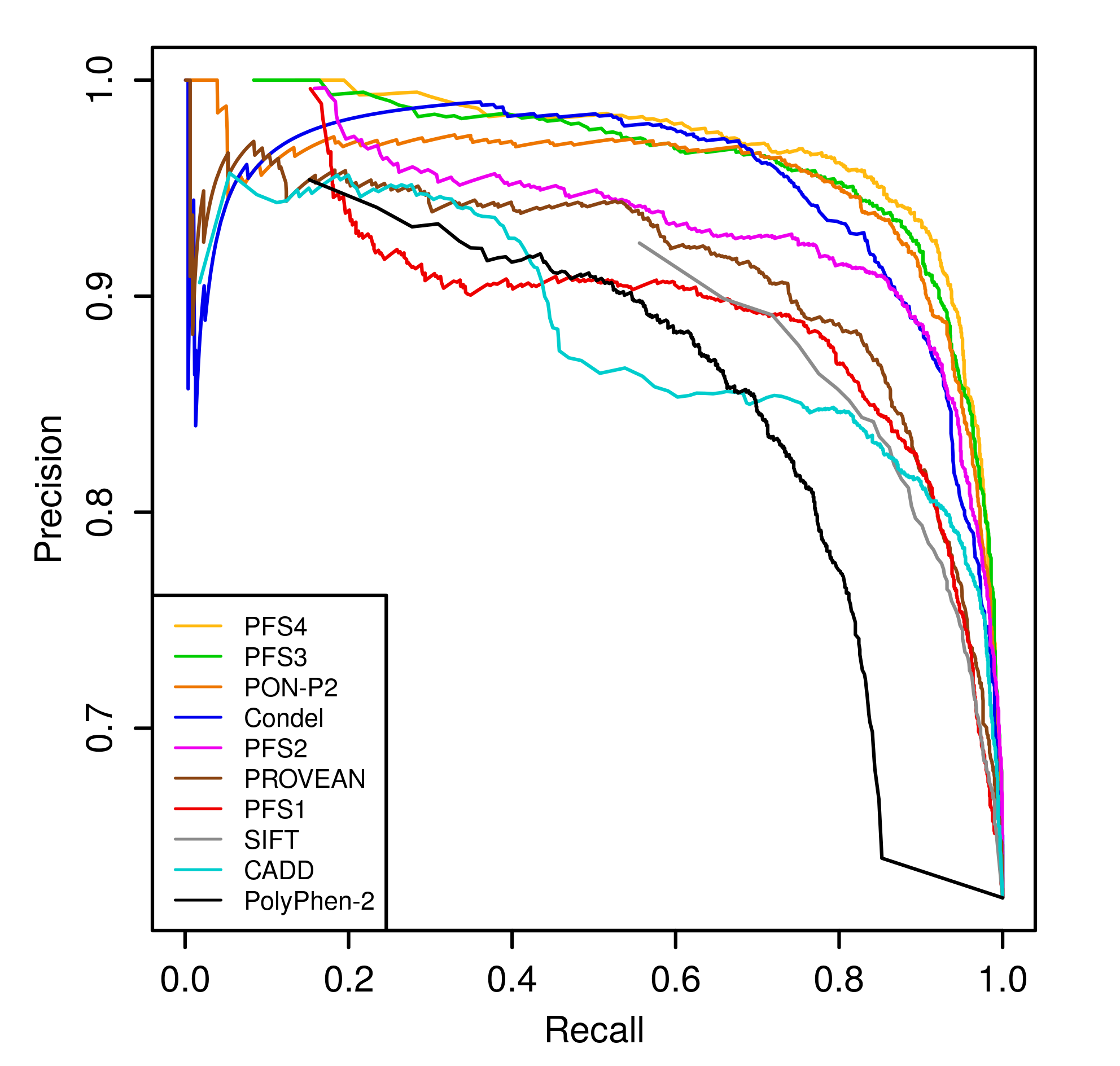


**Supp. Figure S10**. Precision-Recall curves for all methods accessed in this study, as well as Condel, PolyPhen-2, and CADD.

**References**

Le Pera L, Marcatili P, Tramontano A. 2010. PICMI: mapping point mutations on genomes. Bioinformatics 26: 2904–2905.

Weichenberger CX, Blankenburg H, Palermo A, D’Elia Y, König E, Bernstein E, Domingues FS. 2015. Dintor: functional annotation of genomic and proteomic data. BMC Genomics 16: 1081.
